# Probing the substrate binding modes and catalytic mechanisms of BLEG-1, a promiscuous B3 metallo-β-lactamase with glyoxalase II properties

**DOI:** 10.1371/journal.pone.0291012

**Published:** 2023-09-06

**Authors:** Shaw Xian Au, Azyyati Mohd Padzil, Noor Dina Muhd Noor, Hiroyoshi Matsumura, Raja Noor Zaliha Raja Abdul Rahman, Yahaya M. Normi

**Affiliations:** 1 Enzyme and Microbial Technology (EMTech) Research Center, Faculty of Biotechnology and Biomolecular Sciences, Universiti Putra Malaysia, Serdang, Selangor, Malaysia; 2 Department of Cell and Molecular Biology, Faculty of Biotechnology and Biomolecular Sciences, Universiti Putra Malaysia, Serdang, Selangor, Malaysia; 3 Malaysia Genome and Vaccine Institute, National Institutes of Biotechnology Malaysia, Jalan Bangi, Kajang, Selangor, Malaysia; 4 Department of Biochemistry, Faculty of Biotechnology and Biomolecular Sciences, Universiti Putra Malaysia, Serdang, Selangor, Malaysia; 5 College of Life Sciences, Ritsumeikan University, Noji-Higashi, Kusatsu, Japan; 6 Department of Microbiology, Faculty of Biotechnology and Biomolecular Sciences, Universiti Putra Malaysia, Serdang, Selangor, Malaysia; Ahram Canadian University, EGYPT

## Abstract

BLEG-1 from *Bacillus lehensis* G1 is an evolutionary divergent B3 metallo-β-lactamase (MBL) that exhibited both β-lactamase and glyoxalase II (GLXII) activities. Sequence, phylogeny, biochemical and structural relatedness of BLEG-1 to B3 MBL and GLXII suggested BLEG-1 might be an intermediate in the evolutionary path of B3 MBL from GLXII. The unique active site cavity of BLEG-1 that recognizes both β-lactam antibiotics and S-D-lactoylglutathione (SLG) had been postulated as the key factor for its dual activity. In this study, dynamic ensembles of BLEG-1 and its substrate complexes divulged conformational plasticity and binding modes of structurally distinct substrates to the enzyme, providing better insights into its structure-to-function relationship and enzymatic promiscuity. Our results highlight the flexible nature of the active site pocket of BLEG-1, which is governed by concerted loop motions involving loop7+α3+loop8 and loop12 around the catalytic core, thereby moulding the binding pocket and facilitate interactions of BLEG-1 with both ampicillin and SLG. The distribution of (i) predominantly hydrophobic amino acids in the N-terminal domain, and (ii) flexible amino acids with polar and/or charged side chains in both N- and C-termini provide additional advantages to BLEG-1 in confining the aromatic group of ampicillin, and polar groups of SLG, respectively. The importance of these residues for substrates binding was further confirmed by the reduction in MBL and GLXII activities upon alanine substitutions of Ile-10, Phe-57, Arg-94, Leu-95, and Arg-159. Based on molecular dynamics simulation, mutational, and biochemical data presented herein, the catalytic mechanisms of BLEG-1 toward the hydrolysis of β-lactams and SLG were proposed.

## Introduction

Antibiotic resistance (AR) among bacterial pathogens is a serious threat to global healthcare systems. One of the strategies employed by pathogenic bacteria in escaping the action of β-lactam antibiotics is through the production of β-lactamases. β-lactamases hydrolyze the amide bond of the four-membered ring of the antibiotics before they reach the target site of the pathogens. Out of four classes of β-lactamases (class A, B, C, and D), class B metal-dependent β-lactamases or metallo-β-lactamases (MBLs) are of particular concern due to their efficacy in hydrolyzing the last resort antibiotics, carbapenems. Based on the sequence, structure, and metal coordination number of MBLs, they are further divided into B1, B2, and B3 subclasses. Binuclear B1 and B3 MBLs are important drug targets due to their broad activity profile toward penicillins, cephalosporins to carbapenems. B2 MBLs which coordinate one metal ion, on the other hand, hydrolyze only carbapenems specifically [1, 2].

MBLs in general belong to the metallo-hydrolase-like MBL-fold superfamily which consists of a large group of functionally diverse proteins, which includes glyoxalase II (GLXII), N-acyl-L-homoserine lactonase (AHL), flavodiiron protein, ribonuclease (RNase) etc [3]. Members of this superfamily are characterized by the conserved His-Xaa-His-Xaa-Asp-His sequence motif and an αβ/βα structural core domain that accommodate mono- or di-metal ions, such as Zn^2+^, Fe^2+^, Mg^2+^, Mn^2+^, Co^2+^, Ca^2+^ and Ni^2+^ ions for catalytic performance [1, 2]. The additional loop(s) and/or domain(s) to the versatile αβ/βα scaffold near the active site define substrate specificities and catalytic differences among MBL-fold proteins [4]. Interestingly, moonlighting proteins concomitant to native catalytic function and promiscuous β-lactamase activity were discovered in this superfamily, encompassing the kingdoms of bacteria, archaea, and eukarya [5–10]. Examples include MIM-1 and SAM-1 isolated from marine bacteria with potent β-lactamase and AHL activities [5, 6], archaeal MetbaB from *Methanosarcina sp*. which exhibited β-lactamase, RNase, and GLXII activities [7], and the DNA cross-link repair proteins i.e. SNM1A and SNM1B from human cells which have the ability to degrade penicillin in addition to their native activity [8]. Investigations of the primordial representatives in the MBL family such as the highly promiscuous Igni18 with β-lactamase, lipase, lactonase, and phosphodiesterase activities, and the bifunctional PNGM-1 enzyme which acts on β-lactams and unstructured RNAs [9, 10], suggested promiscuous MBLs can serve as good models in tracing the evolutionary origins of MBL; in light of the concept that a specialist enzyme with new function is probably diverged from a generalist enzyme (enzyme with promiscuous functions) during evolution [11]. Despite the substantial foundations of functionally characterized promiscuous MBLs, deeper insights into the structure–function relationships of these enzymes are necessary to delineate the evolutionary trajectories of MBLs.

**Fig 1:**
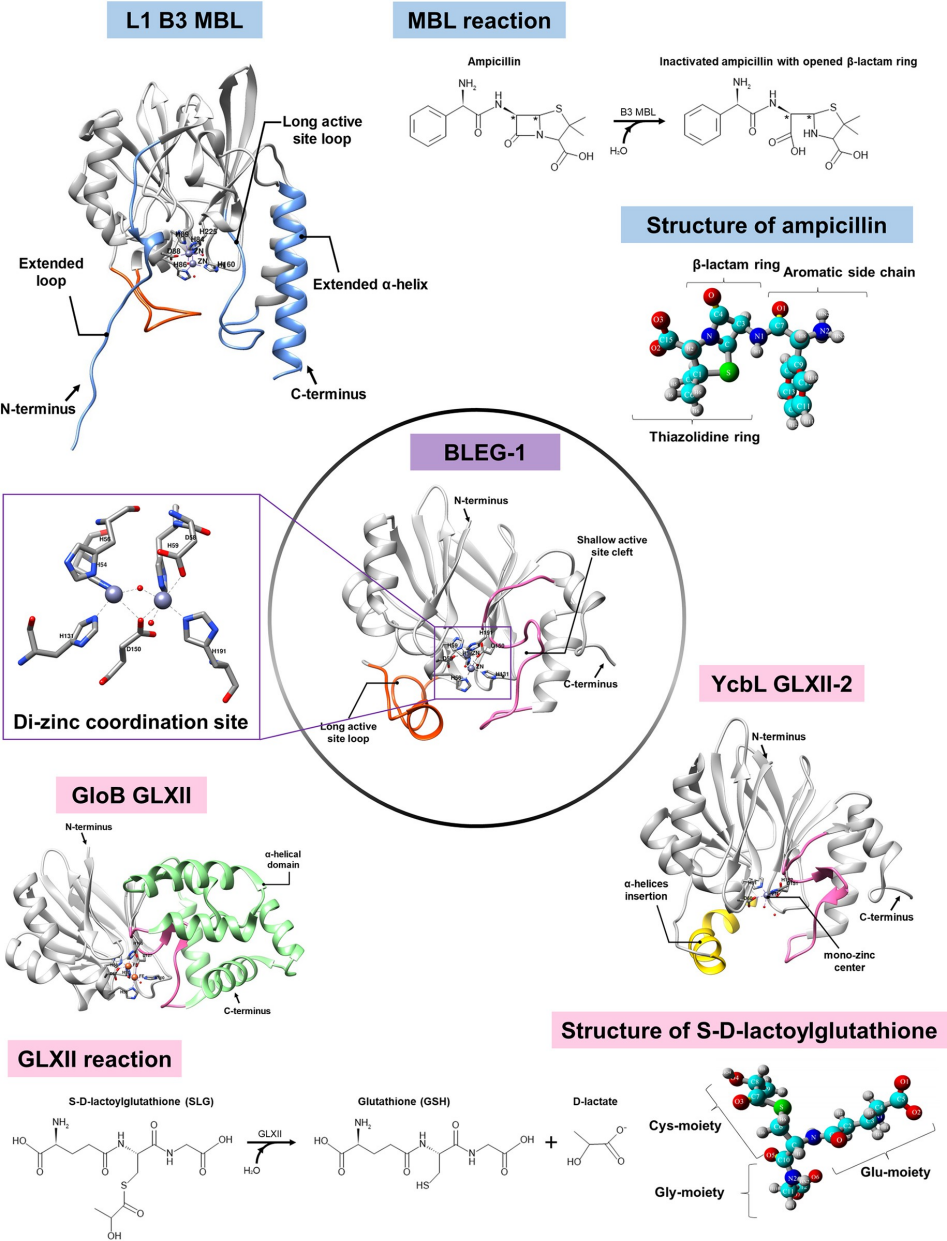
Crystal structures of BLEG-1 (PDB ID: 7EV5), L1 B3 MBL (PDB ID: 1SML), YcbL GLXII-2 (PDB ID: 2XF4), and GloB GLXII (PDB ID: 2QED) demonstrated conservation of the αβ/βα protein fold (gray) among the members of metallo-hydrolase-like MBL-fold superfamily. Most B3 MBLs possess a loop and extended helix at their N- and C-terminus respectively (blue), whereas GLXIIs generally conserved an α-helical domain at their C-terminus (green). The unusual YcbL GLXII (also known as GLXII-2) lacks of the C-terminal α-helical domain and has an α-helix insert that made up its active site cavity in its N-terminal region (yellow). BLEG-1 shows similar active site configuration to both B3 MBLs i.e. a long active site loop in its N-terminal domain (orange), and GLXIIs i.e. a shallow active site cleft at C-terminus (pink). B3 MBLs and GLXII catalyze the hydrolysis of β-lactam antibiotics and S-D-lactoylglutathione (SLG), respectively. The chemical reactions were drawn using MedChem Designer (version 5.5.0.11), while the molecular structure of ampicillin and SLG were generated by YASARA software (version 18.4.24).

BLEG-1 from *Bacillus lehensis* G1 alkaliphile is a promiscuous MBL of B3 subclass that can inactivate almost all classes of β-lactam antibiotics except monobactams, and catalyze the hydrolysis of S-D-lactoylglutathione (SLG) (i.e. the substrate of GLXII enzymes) [12, 13]. Unlike the bacteria-producing B3 MBLs, GLXII is found in both prokaryotes and eukaryotes. It is one of the key enzymes in the glyoxalase system involved in cellular detoxification of methylglyoxal (MG), by catalyzing the hydrolysis of SLG to produce D-lactate and glutathione (GSH). The participation of GLXII in the post-translational modifications and specific metabolic pathways of mammalian cells signified its regulatory role for cell survival [14]. Hence, inhibition of GLXII enzymes had been reported as a potent therapeutic option for cancer or oxidative stress-related diseases [14–19]. Notably, protein structures and substrate binding residues of prokaryotic and eukaryotic GLXIIs are generally conserved, suggesting similar mechanism of action in GLXIIs from different organisms [20]. In bacteria, two types of GLXII have been previously described, which are the (i) common GLXII (eg. *Salmonella typhimurium* GloB) and (ii) GLXII-2, an isozyme of GLXII (eg. *Salmonella enterica* serovar typhimurium YcbL) [20–23]. The sequence, structure, and function of GLXII and GLXII-2 are highly conserved and similar, whereby the lack of an α-helical domain at the C-terminus and the mono-zinc center in GLXII-2 set it apart from common GLXII [20, 22]. Although the structure and phylogeny of B3 MBL and GLXII suggested close relationships between these two protein families, distinct metal coordination geometry and active site topology distinguish their substrate preferences and biochemical properties [4, 24]. Interestingly, despite the conservations of αβ/βα fold and zinc coordination site, BLEG-1 has adopted an active site pocket constituted of structural features of B3 MBL and GLXII which could be crucial for dual substrate binding i.e. a gorge-forming active site loop structure in the N-terminal region that is similarly observed in most B3 MBLs, and a shallow binding cleft at the C-terminal domain which is generally conserved in GLXIIs (Fig 1). Such unique active site configuration could be the possible reason for BLEG-1 to accommodate both β-lactams and SLG, thus displaying enzyme promiscuity [13]. However, previous research based on the static crystallographic structure of BLEG-1 (PDB ID: 7EV5) hampers detailed understanding about the dynamic personalities of the enzyme, which is of more significance in defining its mechanistic basis. The presented study reports on structural dynamicity of BLEG-1 in the binding of ampicillin and SLG, investigated via biophysical and computational approaches. This provides a better insight into the structural dynamicity of BLEG-1, as well as the specific molecular interactions between BLEG-1 and its diverse substrates within the plasticity of the binding groove. Substitutions of key substrate-interacting residues to alanine, followed by enzymatic assays of variants BLEG-1 highlight the actual involvement of these residues in dual substrates recognition and stabilization. Based on the results obtained, the catalytic mechanisms of BLEG-1 toward both ampicillin and SLG are proposed. This research not only unveils the enzymatic promiscuity of BLEG-1 from structural and mechanistic perspectives, but also provides new insights into the reaction mechanism of bacterial GLXII which remains to be elucidated.

## Materials and methods

### Bacterial strains and plasmid vectors

pET-28b(+) (Novagen, Germany) was used as the vector for cloning and expression of variants *BLEG-1* in this study. Bacterial strains, *Escherichia coli* DH5α and BL21(DE3) (Invitrogen, USA) were used for cloning and heterologous production of recombinant variant BLEG-1 proteins, respectively. To produce wild-type (WT) BLEG-1 recombinant protein, *E*. *coli* BL21(DE3) harboring pET28(b)::*bleg-1* described in Au et al. (2021) was used [13].

### Secondary structure and K_d_ analysis via circular dichroism (CD)

Secondary structural changes of WT BLEG-1 upon substrate i.e. ampicillin and S-D-lactoylglutathione (SLG) binding were investigated by comparing the composition of secondary structures of the free enzyme and enzyme-substrate complexes [25, 26] via circular dichroism (CD). Prior to CD experiments, purified WT BLEG-1 protein produced using previously reported procedures [13, 27] was subjected to buffer exchange using 10 mM Na_2_HPO_4_–NaH_2_PO_4_ buffer (pH 7.4) by dialysis at 4°C for 24 hours. The dialyzed protein sample was subsequently filtered by syringe filtration through 0.45 μm cellulose acetate membrane (Sartorius, Germany). To determine the secondary structural elements of WT BLEG-1 with and without substrates, 7.5 μM of WT BLEG-1 and 75 μM of ampicillin and SLG were used, respectively. For determination of the respective K_d_ values of ampicillin and SLG, the following enzyme-substrate molar ratios were used 1:0, 1:1, 1:10, 1:20 and 1:50. CD spectra were collected at 190 nm to 260 nm with a scanning speed of 50 nm/min in a quartz cuvette with pathlength of 0.1 cm (Starna Scientific, USA) using a J-810 spectropolarimeter (Jasco, Japan). All spectra were averaged over three measurements and duly corrected by subtracting the background signal of the buffer solution (for CD analysis of WT BLEG-1 alone) and a mixture of buffer and substrate at above mentioned molar ratio (for CD analysis of BLEG-1 in the presence of substrates). The corrected CD spectra in wavelength range of 195 nm to 240 nm were used to calculate the secondary structural contents of WT BLEG-1 using SpectraManager^TM^ software. In addition, the absorbance of WT BLEG-1 at 223 nm was subjected to non-linear regression analysis and calculation of K_d_ using GraphPad Prism 9.4.1 for Windows, GraphPad Software, San Diego, California USA, www.graphpad.com [28, 29].

### Molecular dynamics (MD) simulations of BLEG-1 and its complexed structures with ampicillin and SLG

MD simulations of the crystal structure of WT BLEG-1 (PDB ID: 7EV5) and its complexed structures with ampicillin and SLG were performed using Yet Another Scientific Artificial Reality Application (YASARA) software (version 18.4.24) [30]. The models of BLEG-1–ampicillin and BLEG-1–SLG complexes used in this study were previously prepared by *in silico* docking by Au et al. (2021) [13]. Prior to MD simulation, all structures were subjected to energy minimization using YASARA software. MD simulation of all structures were performed using NVT ensemble and Amber14 force field under periodic boundaries for 100 ns at a time step of 2.5 fs. The system was solvated in a cubic cell, in which the dimension was 20 Å larger than the protein molecule. Simulation temperature was set according to the temperature for the optimal enzyme activities of BLEG-1 i.e. 303 K for BLEG-1–ampicillin complex and 310 K for BLEG-1–SLG complex [13]. To compare structural changes upon substrate binding, MD simulations of BLEG-1 in its free form were also performed at 303 K and 310 K, respectively. The MD trajectories collected were subsequently analyzed to compute the root mean square deviation (RMSD) and root mean square fluctuation (RMSF) of their Cα in YASARA. Principal Component Analysis (PCA) was used to identify the variability of structural dynamics among BLEG-1 and its substrate complexes, whereby their RMSD and RMSF were included as the variables in PCA using OriginPro 10.0.5.153 software.

To estimate the binding affinities of ampicillin and SLG to BLEG-1 in MD simulations, binding energies of BLEG-1–ampicillin and BLEG-1–SLG complexes were evaluated using molecular mechanics Poisson–Boltzmann surface area (MM/PBSA) method in YASARA software. The calculation of MM/PBSA by YASARA follows the theory of nuclear physics through the equation below [31–33], where more positive binding score indicates better ligand binding [33, 34].

$$Binding\;energy=E_{potReceptor}+E_{solvReceptor}+E_{potLigand}+E_{solvLigand}‐E_{potComplex}‐E_{solvComplex}$$

where E_pot_ is the potential energy contributed by bond, angle, dihedral, planarity, van der Waals, and electrostatic interactions; E_solv_ is the solvation energy which includes polar and non-polar solvation.

Analyses and visualization of MD trajectories, structural changes, enzyme-substrate interactions i.e. hydrogen bonds, hydrophobic interactions, van der Waals forces, distance-time evolution of key enzyme-substrate interactions were performed using both YASARA and UCSF Chimera software (version 1.15) [35]. Based on the MD data, residues which could be significant for substrate interactions of BLEG-1 were identified and targeted for mutagenesis study.

### Genes

The sequence of wild-type (WT) *BLEG-1* retrieved from the National Library of Medicine (NCBI) (https://www.ncbi.nlm.nih.gov/protein/655553190, accessed on September 26^th^, 2021) was subjected to codon optimization for expression in *Escherichia coli* B series using Integrated DNA Technologies (IDT) SciTools (https://sg.idtdna.com/CodonOpt, accessed on October 15^th^, 2021). The codon optimized WT *BLEG-1* sequence was used for the design of *BLEG-1* variants, by substituting the existing codon with GCG which encodes alanine residue at the chosen positions. Following this, the genes of *BLEG-1* variants were commercially synthesized and cloned into pUCIDT-Amp vector by IDT Inc., USA, giving forth pUCIDT-Amp::*I10A*, pUCIDT-Amp::*F57A*, pUCIDT-Amp::*R94A*, pUCIDT-Amp::*L95A*, and pUCIDT-Amp::*R159A* recombinant plasmids.

### Cloning of synthetic variants of *BLEG-1* genes into pET-28b(+) expression plasmid

The above mentioned recombinant plasmids harboring variants *BLEG-1* genes (pUCIDT-Amp::*vBLEG-1* in general) were transformed into competent *E*. *coli* DH5α cells by heat-shock method [36]. The plasmids were extracted from an overnight culture of the positive transformant using NucleoSpin® Plasmid DNA Purification Kit (Machery-Nagel, Germany), based on manufacturer’s instructions. The extracted pUCIDT-Amp::*vBLEG-1* were used as the template for amplification of variant *BLEG-1* via polymerase chain reaction (PCR). For this purpose, 50 μL PCR mixtures containing 2 ng DNA template, 1 × PCRBIO reaction buffer (3 mM MgCl_2_, 1 mM dNTPs, enhancers, and stabilizers), 10 μM forward and reverse primers, and 2.5 U Taq polymerase (PCR Biosystems, UK) were prepared. The forward and reverse primers: 5’-GCGCTAGCATGTTCAAACAAATCCC-3’ and 5’-GTAAGCTTGATTAATACCCAGTCAAAAACG-3’, were specifically designed to incorporate *NheI* and *HindIII* restriction sites to 5’ and 3’ ends of variants *BLEG-1* genes, respectively (indicated by underline). Amplification of variants *BLEG-1* was performed using the following PCR program: 1 cycle of initial denaturation at 95°C for 1 min, followed by 30 cycles of denaturation at 95°C for 15 s, annealing at 52°C for 15 s, extension at 72°C for 40 s, and lastly 1 cycle of final extension at 72°C for 5 min. The 647-bp amplicon was purified using *EasyPure*® PCR Purification Kit (TransGen Biotech, China) based on manufacturers recommendations and subsequently digested by high fidelity (HF) *Nhe*I and *HindIII* restriction enzymes (REs) (New England Biolab, UK). The digested variant *BLEG-1* DNA fragments were ligated to pET-28b(+) vector which was similarly digested with the same REs, using T4 DNA ligase (Thermo Fisher Scientific, USA). The ligation products were transformed to competent *E*. *coli* DH5α cells, whereby the recombinant plasmids i.e. pET-28b(+)::*vBLEG-1* were subsequently extracted for verification via Sanger sequencing (Apical Scientific Sdn. Bhd., Malaysia). Recombinant plasmids carrying the correct gene sequences of *BLEG-1* variants were transformed into competent *E*. *coli* BL21(DE3) cells for protein expression.

### Overexpression and purification of WT and variants BLEG-1

Recombinant WT and variants BLEG-1 were overexpressed in *E*. *coli* BL21(DE3) by isopropyl β-D-1-thiogalactopyranoside (IPTG)-induction and subsequently purified via Ni-NTA affinity chromatography using the methods described by Au et al. (2021, 2022) [13, 27]. The proteins were analyzed by SDS-PAGE (12%) using Laemmli system [37] and quantified by Bradford assay [38], using Bradford reagent (VWR International, USA) with bovine serum albumin as standard.

### Enzymatic assays of WT and variants BLEG-1

Enzyme activity assays were conducted in a 1.4 mL Quartz cuvette (Starna Scientific, USA) with a Varian Cary 50 Bio UV-Visible Spectrophotometer (Agilent Technologies, USA). MBL and GLXII activities of BLEG-1 were tested using ampicillin (GOLDBIO, Philippines) and SLG (Sigma-Aldrich, USA) as respective substrates based on the methods described by Au et al. (2021) with slight modifications [13]. Both assays were carried out in 1 mL volume of 20 mM Na_2_HPO_4_–NaH_2_PO_4_ buffer, pH 7 containing 1 μM of purified WT or variant BLEG-1 protein, 20 μg/mL bovine serum albumin, 100 μM ZnSO_4_, and 100 μM ampicillin or SLG. MBL and GLXII assays were conducted at 30°C and 37°C, respectively. The reaction was monitored by recording the changes in absorbance (∆A_235nm_ and ∆A_240nm_ for MBL and GLXII assays, respectively) at the interval of 1 minute for a total duration of 6 minutes. All assays were performed in triplicates.

## Results

### Circular dichroism (CD) suggested BLEG-1 promiscuity in substrate interactions

**Fig 2:**
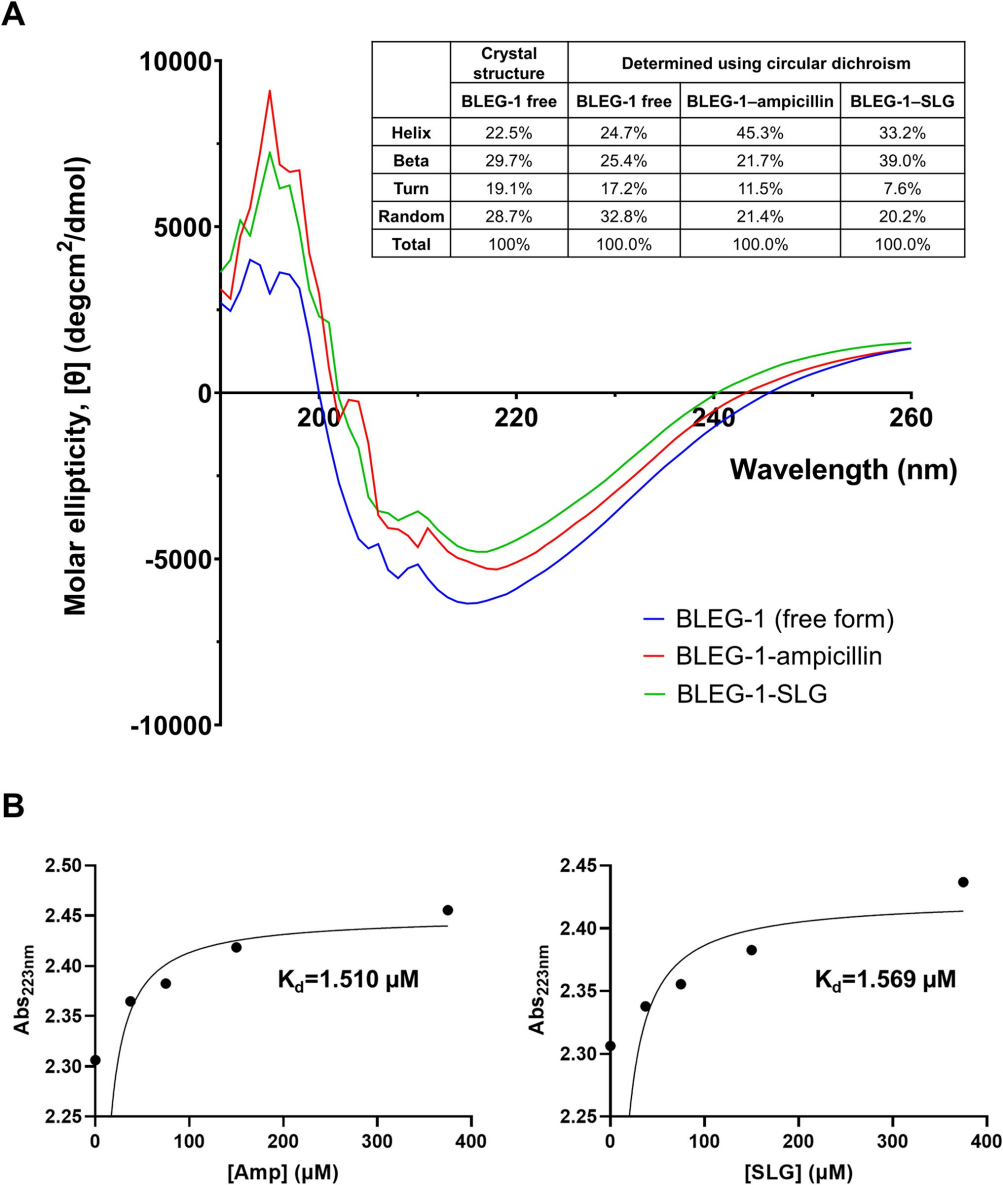
Substrate binding properties of BLEG-1 determined using CD spectroscopy. (A) Changes of secondary structure upon ampicillin and SLG binding. (B) Determination of dissociation constants (K_d_) of ampicillin (left) and SLG (right) in BLEG-1 using non-linear regression analysis.

Previous docking analyses proposed that BLEG-1 possesses an active site configuration which could accommodate both ampicillin and S-D-lactotylglutathione (SLG), suggesting this may be the reason for its enzymatic promiscuity [13]. In this study, changes in secondary structures of BLEG-1 upon substrate binding were investigated using CD. Firstly, CD results indicate that the secondary structural compositions of free WT BLEG-1 are in agreement with the crystal structure of the protein (PDB ID: 7EV5) (Fig 2A), signifying the reliability of the CD data and analysis. Secondly, the results also showed that substrate-free and substrate-bound states of BLEG-1 yielded different CD signals at wavelength range of 190 nm to 260 nm, indicating conformational changes of the protein upon the addition of ampicillin and SLG. More helices were formed in BLEG-1 upon interaction with ampicillin, whereas formation of both helices and sheets was observed upon BLEG-1 interaction with SLG (Fig 2A).

Computation of the dissociation constants (K_d_) of ampicillin and SLG based on CD data revealed that the K_d_ values of these substrates were comparable (Fig 2B), indicating BLEG-1 has similar affinity toward ampicillin and SLG. It is also worthy to note that the K_d_ of ampicillin determined in this study (K_d_ = 1.51 μM) is highly similar to that determined previously using nano-isothermal titration calorimetry (ITC) (K_d_ = 1.47 μM) by Selvaraju et al. (2020) [39], suggesting the reliability of the experiment and data obtained. It is important to add that although nano-ITC of BLEG-1–SLG complex was initially conducted in the present study, the expected heat profile was unattainable, most probably due to heat cancelation arising from the formation of products (S1 Fig). Hence, CD experiments and analysis have provided an alternative means in obtaining reliable K_d_ values of the substrates involved.

### BLEG-1 possess rigid protein core and mobile loop structures

Molecular dynamics (MD) simulation of BLEG-1–ampicillin and BLEG-1–SLG complexes were executed at different temperatures i.e. 30°C and 37°C respectively, as these are the optimal temperatures for the hydrolytic activities of the enzyme toward ampicillin and SLG. For accurate comparison of the dynamic entities of BLEG-1 and its substrate complexes, MD simulations of BLEG-1 (free form) were also performed at both temperatures.

**Fig 3:**
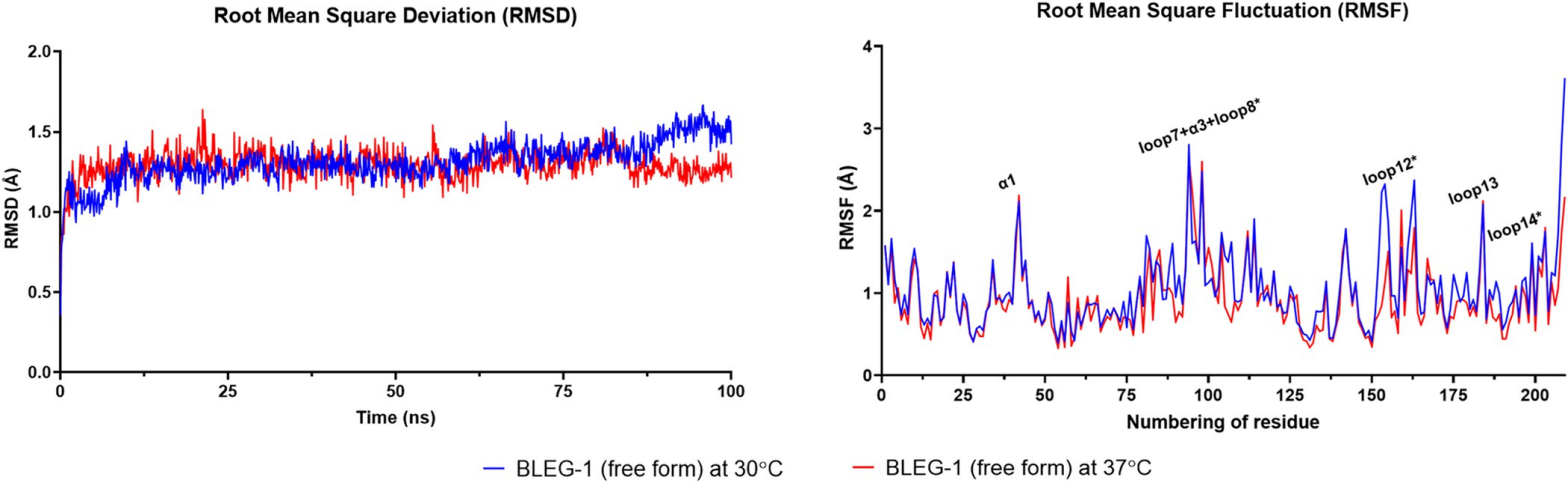
MD simulation of BLEG-1 (free form). Root mean square deviation (RMSD) (left) and root mean square fluctuation (RMSF) (right) of BLEG-1 (free form) at 30°C (blue line) and 37°C (red line). Active site loops are labelled with asterisk (*) in the RMSF plot.

Root mean square deviation (RMSD) and root mean square fluctuation (RMSF) analyses of the Cα of unbound BLEG-1 showed comparable results at 30°C and 37°C (Fig 3), suggesting BLEG-1 exhibited similar conformational stability and flexibility in both systems. Throughout 100 ns simulation time, the enzyme structure remained stable as evident from its stable RMSD value. Flexible regions of BLEG-1 were subsequently identified based on the structural fluctuation of each amino acid. The RMSF values of most residues of BLEG-1 were less than 1 Å, whereas the residues positioned on the active site loops i.e. loop7+α3+loop8 (Arg-81–Asp-107) and loop12 (Asp-150–Asp-166), and certain solvent-exposed structures i.e. α1 (Ala-33–Arg-42) and loop13 (Leu-179–Thr-185) possess RMSF values of more than 2 Å (Fig 3, right). This indicates that the overall structure of BLEG-1 was rigid, while the active site loops and some solvent-exposed structures behaved flexibly. Notably, one of the active site loops i.e. loop14 (Gly-190–Thr196) was relatively stable than loop7+α3+loop8 and loop12, due to the formation of intramolecular hydrogen bonds between loop14 and its neighboring β11 strand (Ile-186–Ser-189).

### Binding of ampicillin induced structural flexibility of BLEG-1

**Fig 4:**
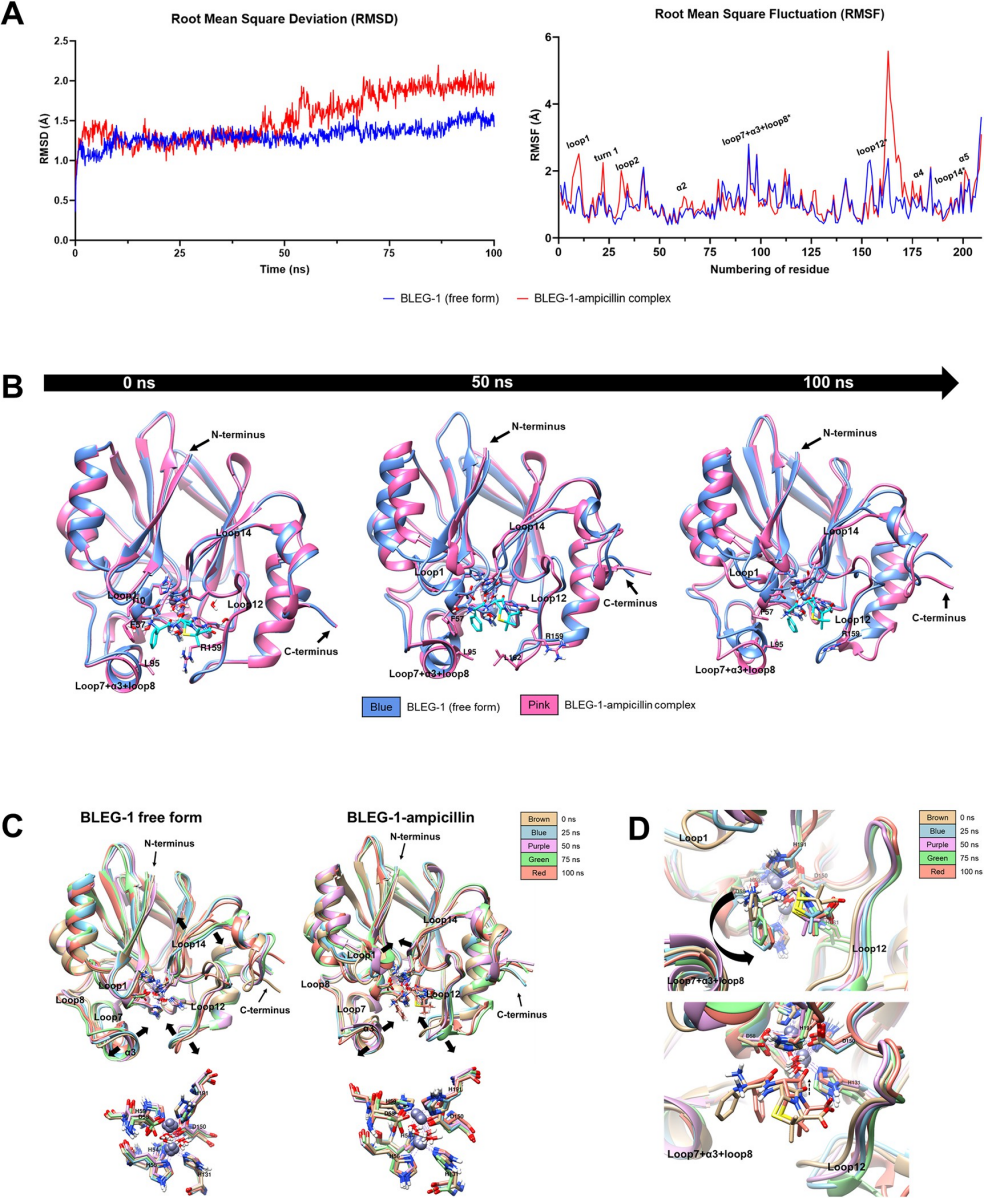
MD simulation of BLEG-1–ampicillin complex. (A) Root mean square deviation (RMSD) (left) and root mean square fluctuation (RMSF) (right) analyses of BLEG-1 (free form) (blue line) and BLEG-1–ampicillin complex (red line). Active site loops are labelled with asterisk (*) in the RMSF plot. (B) Structural superimpositions of BLEG-1 (free form) and BLEG-1–ampicillin complex at 0, 50, and 100 ns. (C) Superimpositions of the overall structure (top) and zinc coordination site (bottom) of BLEG-1 (free form) (left) and BLEG-1–ampicillin complex (right) at 0, 25, 50, 75, and 100 ns. (D) Structural superimpositions of ampicillin molecule at 0, 25, 50, 75, and 100 ns (top); initial (at 0 ns) and final (at 100 ns) orientations of ampicillin (bottom).

BLEG-1 (free form) and BLEG-1–ampicillin complex possessed similar RMSD of 1.5 Å from 0 to 50 ns, indicating that BLEG-1 exhibits similar structural stability upon ampicillin binding for the first 50 ns. After 50 ns, BLEG-1–ampicillin complex showed an increase in RMSD, approximately 0.5–1 Å higher than that of BLEG-1 (free form), and achieved equilibrium beyond 75 ns (Fig 4A, left). This suggests that the binding of ampicillin in BLEG-1 may have slightly destabilized the protein structure after 50 ns. Further investigation of the MD trajectories revealed that structural destabilization may be due to the conformational changes of BLEG-1 caused by minor orientation of ampicillin.

Upon interaction with ampicillin, loop12 segment of BLEG-1 that is located far from the metal center (Arg-159–Asp-166) and its neighboring α4 (His-167–Ile-178) exhibited drastic increase in RMSF values. This suggests that interaction with ampicillin induced more flexibility to these structures. In contrast, loop12 segment which is closer to the catalytic core (Asp-150–Gly-158) seemed to be stabilized in BLEG-1–ampicillin complex, as evident from its lower RMSF value compared to the free enzyme (Fig 4A, right). Other two active site loops i.e. loop7+α3+loop8 and loop14 possessed comparable RMSF values in both free and ampicillin complexed states, indicating similar inherent flexibility of the loops with or without interaction with ampicillin. On the other hand, some solvent-exposed structures of BLEG-1, such as loop1 (Gly-8–Gln-11), turn1 (Asn-19–Lys-22), loop2 (Asp-28–Asp-32), α2 (Val-63–Phe-70), α4 (His-167–Ile-178) and α5 (Ile-197–His-203), which were initially stable in the free enzyme, gained dynamicity as BLEG-1 interacts with ampicillin, as exhibited by their elevated RMSF values (Fig 4A, right).

### Flexible loops adjusted the orientation of ampicillin in BLEG-1

Binding of ampicillin in BLEG-1 induced conformational changes on the active site loops i.e. loop1, loop7+α3+loop8, loop12 and loop14 and the solvent-exposed α-helices, whereas the central β-sheets exhibited minor shifts in general (Fig 4B). This indicates that the interactions between BLEG-1 and ampicillin mainly influenced the dynamics of loop segments and solvent-exposed structures but did not exert much changes on the rigid core structure of BLEG-1. In addition, no notable structural shifts were observed on the zinc coordinating residues as BLEG-1 gained dynamicity or bound to ampicillin, suggesting that the architecture of zinc coordination site is well conserved by the second shell ligands through hydrogen bonds network.

As BLEG-1 binds to ampicillin, loop motions were observed in loop1 due to the formation of hydrogen bond between Gln-12 (loop1) and Gly-192 (loop14). Such interaction brought loop14 and the aromatic side chain (ASC) of ampicillin to close proximity, forming van der Waals interactions between ND-His-191 (i.e. zinc coordinating residue) and O1-ampicillin that further stabilize the conformation of ampicillin in the active site. Loop14 and His-191 gain stabilization in turn from such interactions (Fig 4C). The other two active site loops which flank the zinc coordination site i.e. loop7+α3+loop8 and loop12, demonstrated flexibility despite ampicillin binding, and show their involvement in facilitating the binding and orientation of ampicillin molecule in BLEG-1 (Fig 4C). At the beginning, loop1 and loop7+α3+loop8 in the N-terminal domain of BLEG-1 interacted with the ASC of ampicillin in the vicinity of metal center. Subsequent movements of loop1 and loop7+α3+loop8 orientated the ASC of ampicillin upwards and projected it toward the solvent-exposed region (Fig 4D, top). In the C-terminal domain, loop12 and loop14 stabilized the penam ring of ampicillin by interacting with the thiazolidine ring by His-131 and Arg-159 (loop12); this explains the stabilization of partial segment of loop12 which sat adjacent to the zinc coordination site in BLEG-1–ampicillin complex. Conformational regulation of ampicillin in BLEG-1 brought the carbonyl carbon of the β-lactam ring approximately 1 Å closer to the catalytic water (Fig 4D, bottom), and maintained their distance below 9 Å (i.e. 3–6 Å), which is the acceptable distance for nucleophilic attack as reported by Zhen et al. (2014) [40, 41]. In addition, the final orientation of ampicillin (at 100 ns) in BLEG-1 shows similarity to the binding mode of penicillin in the crystal structure of L1 B3 MBL–penicillin (PDB ID: 6U0Z) (S2 Fig) [42]. The presented MD data is in line with our previous postulation based on docking results [13], whereby the movement of loop1 and loop7+α3+loop8 enabled them to serve as the “doorkeeper” structure in governing the entrance of β-lactam antibiotics to the active site pocket.

### Specific molecular interactions between BLEG-1 and ampicillin

**Fig 5:**
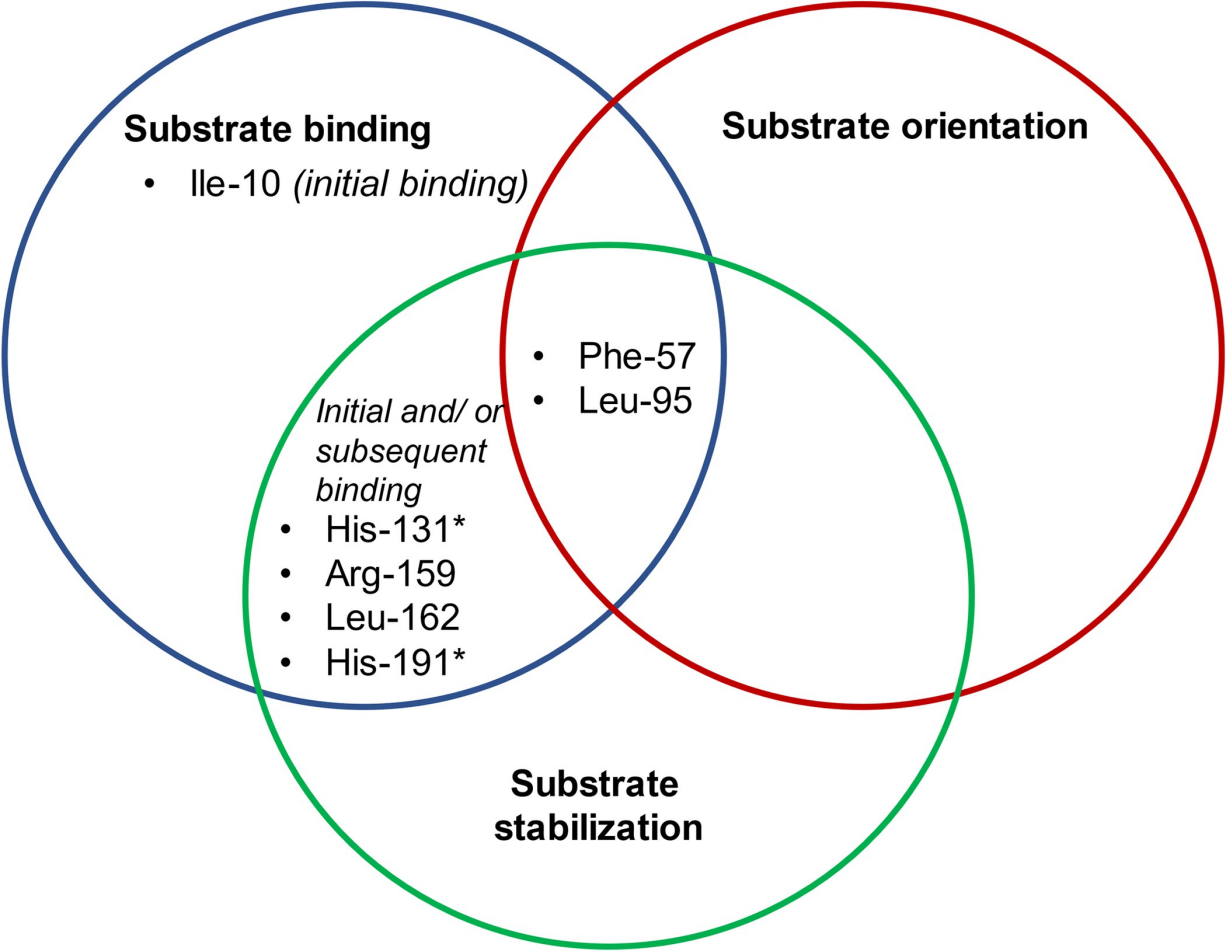
The role(s) of active site residues for the interactions of ampicillin in BLEG-1 postulated based on MD analyses. Zinc coordinating residues are labelled with asterisk (*).

Previous docking experiment predicted the best binding pose of ampicillin to BLEG-1 and ampicillin-interacting residues in the static enzyme [13]. In the current study, MD simulations were used to further analyze the molecular interactions of BLEG-1 and ampicillin in explicit solvent. Results of MD simulation of BLEG-1–ampicillin complex agrees to those of docking analysis, whereby the residues in the N-terminal domain were significant in binding the ASC of ampicillin while the residues in C-terminal region interacted with the penam ring that is composed of β-lactam ring and thiazolidine ring (Table 1). The key interactions between BLEG-1 and ampicillin are the consistent formation of hydrogen bonds with the carboxylate group of thiazolidine ring by His-131 (zinc coordinating residue) and Arg-159 (loop12) in the C-terminal domain throughout 100 ns simulation period. Such interactions maintained the orientation of the penam ring of ampicillin near the zinc coordination site of BLEG-1, in which the carbonyl carbon on the β-lactam ring was further stabilized through the formation of hydrogen bond by Wat2 (apical water). Residues in the N-terminal domain i.e. Phe-57 (η1) and Leu-95 (loop7+α3+loop8) on the other hand, are crucial for binding, orientation and stabilization of the ASC of ampicillin via hydrophobic contacts. Additionally, stacking interactions were established between the phenyl groups of Phe-57 and ampicillin, suggesting its role in strengthening the binding of the ASC of ampicillin in BLEG-1. The accommodation and stabilization of ampicillin was also mediated by other residues i.e. Ile-10 (loop1) and Leu-162 (loop12). Ile-10 was involved in the initial binding of ampicillin, while Leu-162 (loop12) bound and stabilized the ampicillin molecule as the ASC shifted to the vicinity of loop12 during substrate orientation. The interactions by Ile-10, Phe-57, Leu-95 and Leu-162 thereby facilitated conformational adjustment and stabilization of ampicillin in dynamic environment. Detailed interactions of ampicillin with BLEG-1 are outlined in Table 1 and the role of ampicillin-interacting residues were further classified in Fig 5.

**Table 1:** Interactions of ampicillin in BLEG-1 at dynamic state.

Ampicillin interacting residue	Position	Type of interactions	Substructure of ampicillin that interacts with BLEG-1
Ile-10	Loop1	• Hydrophobic interactions	• Phenyl ring of aromatic side chain
Phe-57	η1	• Stacking interactions • Hydrophobic interactions	• Phenyl ring of aromatic side chain
Leu-95	α3	• Hydrophobic interactions	• Phenyl ring of aromatic side chain
Arg-159	Loop12	• Hydrogen bond • Hydrophobic interactions	• -COOH of thiazolidine ring
Leu-162	Loop12	• Hydrophobic interactions	• Phenyl ring of aromatic side chain
His-131	Loop10	• Hydrogen bond • Electrostatic interactions	• -COOH of thiazolidine ring
His-191	Loop14	• Electrostatic interactions	• -C = O of aromatic side chain
Wat2	-	• Hydrogen bond	• -C = O of β-lactam ring; -C = O of aromatic side chain
Zn2	-	• Electrostatic interactions	• -C = O of aromatic side chain

### Time evolution of distance between ampicillin and key interacting residues of BLEG-1

**Fig 6:**
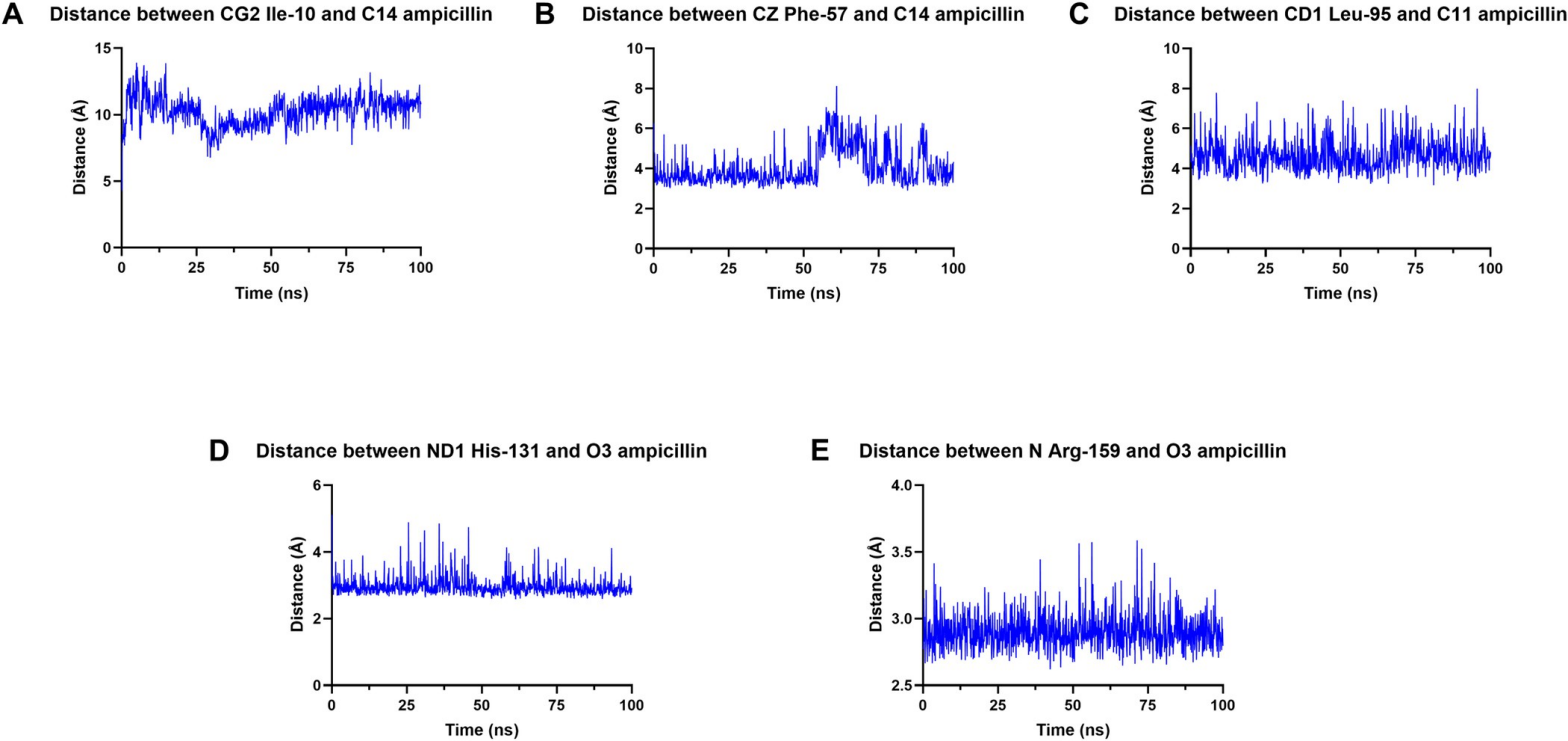
Time evolution of distance between ampicillin and key interacting residues which includes (A) Ile-10, (B) Phe-57, (C) Leu-95, (D) His-131, and (E) Arg-159.

As the hydrophobic environment in the N-terminal region of B3 MBLs had been proposed to be crucial for the interactions of β-lactam antibiotics with hydrophobic substituent [43–45], while the thiazolidine ring of β-lactam could be stabilized via hydrogen bond(s) by polar residue(s) in the C-terminal domain [43], Ile-10, Phe-57, Leu-95, His-131, and Arg-159 of BLEG-1 could play significant role in binding the ampicillin to the enzyme active site. Hence, time evolution of distance between these residues and ampicillin were investigated. Fig 6C–6E show that the distances of ampicillin from Leu-95, His-131 and Arg-159 remained consistent throughout 100 ns period, suggesting their importance for the binding and stabilization of ampicillin molecule. On the other hand, Ile-10 that is involved in the recruitment of ampicillin at the initial stage of the binding event demonstrated an increase in its distance from the ASC of ampicillin after 5 ns (Fig 6A), as a consequence of loop motions mediated by the hydrogen bond between Gln-12 (loop1) and Gly-192 (loop14), which eventually caused the ampicillin to drift away from Ile-10. For Phe-57. Although there was an increase and fluctuation of distances between the residue and ampicillin from 50 to 75 ns due to minor adjustment of substrate conformation, the distances decreased and stabilized after 75 ns, showing similar values as the first 50 ns where uniformity was achieved (Fig 6B).

### Binding of SLG stabilized most flexible structures in BLEG-1

**Fig 7:**
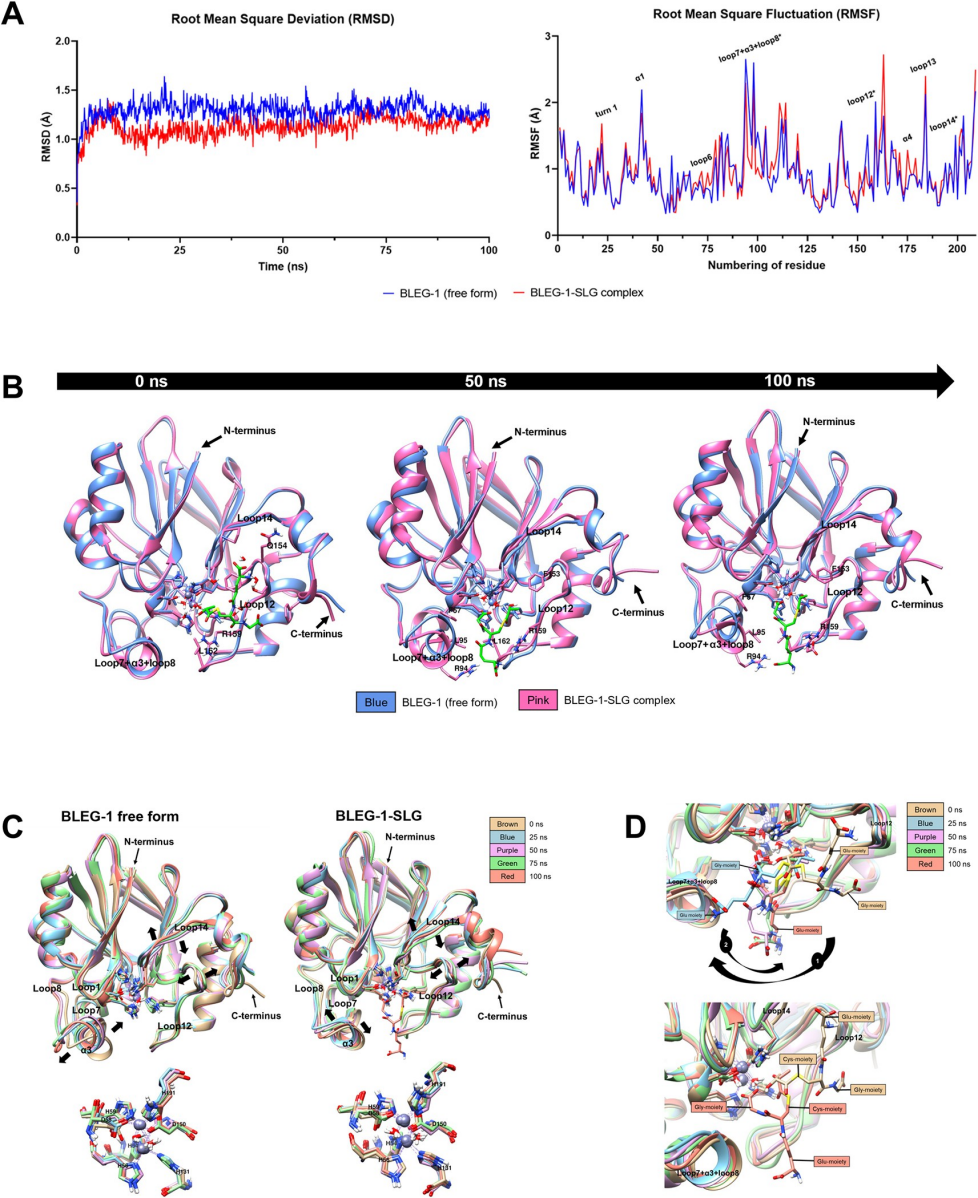
MD simulation of BLEG-1–SLG complex. (A) Root mean square deviation (RMSD) (left) and root mean square fluctuation (RMSF) (right) analyses of BLEG-1 (free form) (blue line) and BLEG-1–SLG complex (red line). Active site loops are labelled with asterisk (*) in the RMSF plot. (B) Structural superimpositions of BLEG-1 (free form) and BLEG-1–SLG complex at 0, 50, and 100 ns. (C) Superimpositions of the overall structure (top) and zinc coordination site (bottom) of BLEG-1 (free form) (left) and BLEG-1–SLG complex (right) at 0, 25, 50, 75, and 100 ns. (D) Structural superimpositions of SLG molecule at 0, 25, 50, 75, and 100 ns (top); initial (at 0 ns) and final (at 100 ns) orientations of SLG (bottom).

In general, the RMSD values of BLEG-1–SLG complex was approximately 0.5 Å lower than that of the free enzyme, indicating that BLEG-1 exhibited greater stability as it bound to SLG. However, higher RMSD values of BLEG-1–SLG complex were observed in the first 10 ns, which may be contributed by the orientation of SLG molecule in the active site pocket at initial dynamic phase (Fig 7A, left).

Interactions of BLEG-1 with SLG stabilized most flexible structures in BLEG-1, which are loop7+α3+loop8, loop12 and α1, as indicated by the decline in RMSF values in these regions. However, loop13, a flexible structure on the protein surface gained more fluctuation upon interaction with SLG. Some solvent-exposed structures which demonstrated stability in BLEG-1 (free form) such as turn1 (Asn-19–Lys22), loop6 (Ser-71–Val-74), α4 (His-167–Ile-178), were also destabilized by SLG, as shown in their elevated RMSF values (Fig 7A, right).

### Mobile loops of BLEG-1 were stabilized after mediating the orientation of SLG

The interactions of BLEG-1 and SLG induced major structural displacements on the N-terminal active site loop i.e. loop7+α3+loop8; this loop demonstrated large amplitude of mobility in BLEG-1 (free form), but it was stabilized upon SLG binding (Fig 7B and 7C). On the other hand, the active site loops on the C-terminal domain i.e. loop12 and loop14, as well as some structures on protein surfaces exhibited minor conformational changes. There were no noticeable structural shifts on the central β-sheets and zinc coordinating residues. Therefore, SLG binding affects the mobility of loops and solvent-exposed structures, while the protein core structure remained stable and intact.

The concatenated trajectories of BLEG-1 (free form) show the flexibility of loop7+α3+loop8, loop12 and loop14 (Fig 7C, left). These loops played crucial role in the accommodation, orientation, and stabilization of SLG in BLEG-1. Previous docking analysis proposed that the SLG molecule is solely bound by the residues in the C-terminal domain of BLEG-1, in which the Gly- and Glu-moieties of SLG were accommodated by loop12 and loop14 respectively [13]. As BLEG-1 gained dynamicity, the mobile active site loops facilitated the positioning or adjustment of SLG in the binding groove, shifting the SLG molecule from the C-terminal region to the center, stabilized by residues in both N- and C-termini upon substrate positioning (Fig 7D, top). The final orientation of SLG revealed that its Gly-moiety was accommodated in the N-terminal region of BLEG-1 while the Glu-moiety projected outward the catalytic core and stabilized by the residues on loop7+α3+loop8 and loop12. The conformational changes of SLG reduced the mobility of loop7+α3+loop8 and loop12 flanking the binding pocket and conferred structural stabilization on BLEG-1 (Fig 7C, right). Such structural changes and substrate orientation were reflected in the RMSD of BLEG-1–SLG complex, in which the values decreased upon the orientation of SLG after 10 ns simulation period. Worth noting is that the Cys-moiety of SLG was still interacting with residues near the zinc coordination site of BLEG-1 albeit the conformational adjustment. The carbonyl carbon of the Cys-moiety which would be attacked by the nucleophile, was brought approximately 1 Å toward the catalytic water of BLEG-1, where their distance was maintained in the range of 3–6 Å throughout the simulation (Fig 7D, bottom). The final binding mode of SLG in BLEG-1 demonstrated resemblance to the accommodation of S-(N-hydroxy-N-bromophenylcarbamoyl)glutathione (a substrate analogue of SLG; abbreviated as HBPC-GSH) in human GLXII (hGLXII) (PDB ID: 1QH5), with the exception that the Gly-moiety of HBPC-GSH interacted with the C-terminal α-helical domain of hGLXII (S3 Fig), which is generally present in GLXIIs but not in BLEG-1 [46].

### Specific molecular interactions between BLEG-1 and SLG

**Fig 8:**
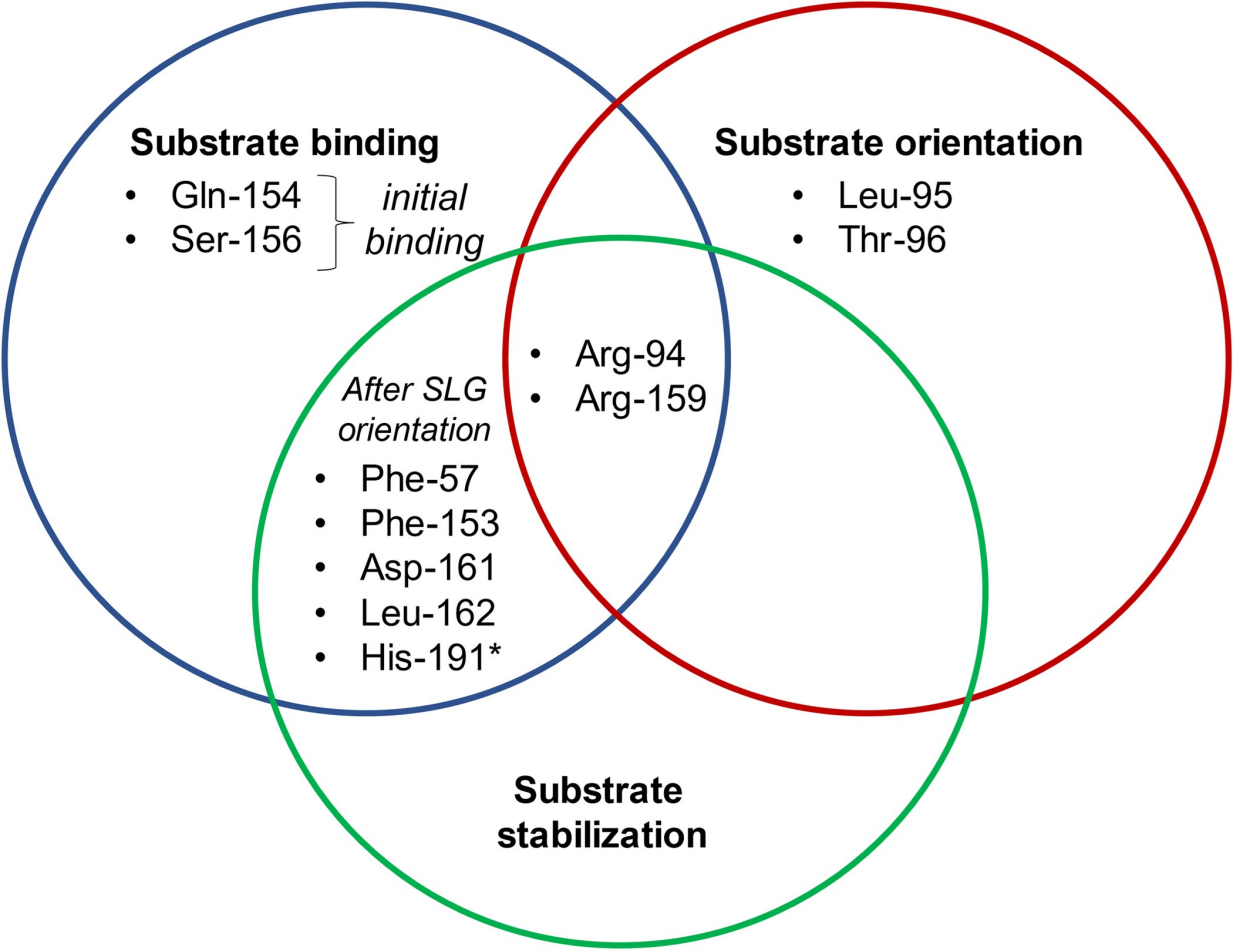
The role(s) of active site residues for the interactions of SLG in BLEG-1 postulated based on MD analyses. Zinc coordinating residue is labelled with asterisk (*).

Apart from the SLG binding residues predicted by docking analysis [13], Phe-57, Arg-94, Leu-95, Thr-96, Asp-161, and Leu-162 of BLEG-1 also showed interactions with SLG, due to the conformational adjustment of SLG molecule at dynamic state. Table 2 demonstrates the molecular interactions between BLEG-1 and SLG throughout MD simulation. Arg-94 (loop7+α3+loop8) and Arg-159 (loop12) in the N- and C-terminal region respectively revealed significant interactions with Glu-moiety of SLG, whereby the formation of hydrogen bonds occurred regularly throughout 100 ns. Arg-159 with its flexible side chain, which initially interacted with SLG in the C-terminal domain together with Gln-154 and Ser-156 through hydrogen bonding for the first 5 ns, facilitated the orientation of SLG and shifted it to the center of the active site. Arg-94 in turn, interacted and stabilized the orientation of SLG molecule cooperatively with Arg-159 by interacting with the Glu-moiety of SLG. In addition to the arginine residues, conformational displacement of SLG was also mediated by Leu-95 and Thr-96 positioned on loop7+α3+loop8 via temporal hydrogen bonds around 18–25 ns, as well as additional hydrophobic interactions by Leu-95. Upon SLG orientation, further stabilization of the molecule was attained involving the following residues in the active site pocket: Gly-moiety (Phe-57 and Leu-162); Cys-moiety (Phe-153 and His-191); Glu-moiety (Asp-161). Beside the interactions by amino acids, Cys- and Gly-moieties of SLG were further stabilized in the metal center of BLEG-1 by catalytic water and dizinc ions via hydrogen bonding and electrostatic attractions respectively. This enabled the carbonyl carbon of Cys-moiety to be retained in the zinc coordination site for nucleophilic attack and stabilization of the Gly-moiety during the formation of enzyme-substrate (ES) transition state. Based on the MD simulation of BLEG-1–SLG complex, the roles of SLG-interacting residues are duly classified in Fig 8.

**Table 2:** Interactions of SLG in BLEG-1 at dynamic state.

SLG interacting residue	Position	Type of interactions	Substructure of SLG that interacts with BLEG-1
Phe-57	η1	• Hydrophobic interactions	• Carbon chain of Gly-moiety
Arg-94	α3	• Hydrogen bond	• -COOH and -NH_2_ of Glu-moiety
Leu-95	α3	• Hydrogen bond • Hydrophobic interactions	• -NH_2_ of Glu-moiety • Carbon chain of Glu- and Gly-moieties
Thr-96	α3	• Hydrogen bond	• -NH_2_ of Glu-moiety
Phe-153	Loop12	• Hydrophobic interactions	• Carbon chain of Cys-moiety
Gln-154	Loop12	• Hydrogen bond	• -NH_2_ of Glu-moiety
Ser-156	Loop12	• Hydrogen bond	• -NH_2_ and -C = ONHR of Glu-moiety
Arg-159	Loop12	• Hydrogen bond • Hydrophobic interactions	• -COOH and -NH_2_ of Glu-moiety; -COOH of Gly-moiety • Carbon chain of Cys-moiety
Asp-161	Loop12	• Hydrogen bond • Hydrophobic interactions	• -NH_2_ of Glu-moiety • Carbon chain of Glu-moiety
Leu-162	Loop 12	• Hydrophobic interactions	• Carbon chain of Gly-moiety
His-191	Loop14	• Hydrophobic interactions	• Carbon chain of Cys-moiety
Wat1	-	• Hydrogen bond	• -COOH of Gly-moiety
Wat2	-	• Hydrogen bond	• -COOH of Cys-moiety
Zn1	-	• Electrostatic interactions	• -COOH of Gly-moiety
Zn2	-	• Electrostatic interactions	• -COOH of Cys- and Gly-moieties

### Time evolution of distance between SLG and key interacting residues of BLEG-1

Out of all interactions observed in the MD simulations of BLEG-1–SLG, Phe-57, Arg-94, Leu-95, and Arg-159 are of most interest. In BLEG-1, Arg-159 is structurally aligned to Lys-142 in *Arabidopsis* GLXII, which its involvement in SLG binding had been evidenced through site-directed mutagenesis [47]. Hence, it is of interest to verify the actual involvement of Arg-159 in the accommodation of SLG in BLEG-1. Unlike common GLXIIs, BLEG-1 lacks the C-terminal α-helical domain that is important for the interactions of SLG substrate [46]. Therefore, residues in the N-terminal domain of BLEG-1 which portrayed possible interactions with SLG in the MD simulation, including (i) Phe-57 that stabilizes the Gly-moiety of SLG, as well as (ii) Arg-94 and (iii) Leu-95 which interacts with the Glu-moiety of SLG are worth to be considered for further investigations.

**Fig 9:**
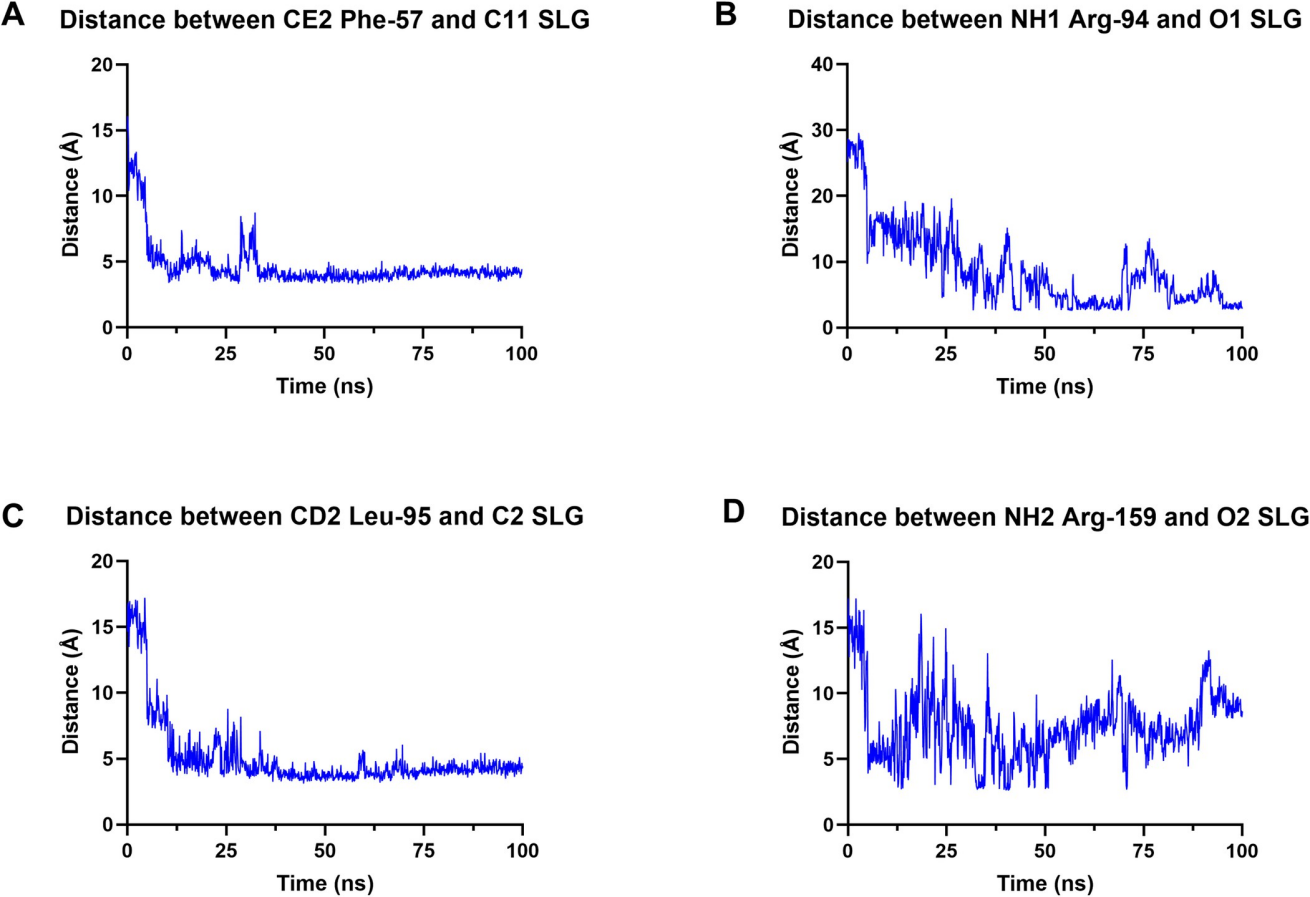
Time evolution of distance between SLG and key interacting residues which includes (A) Phe-57, (B) Arg-94, (C) Leu-95, and (D) Arg-159.

Fig 9 demonstrates that the distances of Phe-57, Arg-94, Leu-95 and Arg-159 of BLEG-1 from the SLG molecule drastically decreased after 10 ns, showing agreement to the conformational adjustment of SLG described in the above section. Phe-57 and Leu-95 generally portrayed consistent changes in their distances from the Gly- and Glu-moieties of SLG respectively throughout the simulation, except for the slight fluctuation between 25–30 ns which is caused by the orientation of SLG from the N- to C-terminal region of BLEG-1 at that period. In contrast, distance-time evolution of Arg-94 and Arg-159 suggested fluidity in interactions between these residues and SLG. This is perpetuated by the long, flexible side chains of these arginine residues when interacting with the Glu-moiety of SLG. As the Glu-moiety of SLG moves at the “entrance” of the active site pocket of BLEG-1, it is interacted by the arginine residues at random initially, whereby Arg-159 bound more regularly for the first 50 ns, while Arg-94 interacted more often subsequently.

### Principal component analysis (PCA) and MM/PBSA binding energy analysis of BLEG-1–ampicillin and–SLG complexes

**Fig 10:**
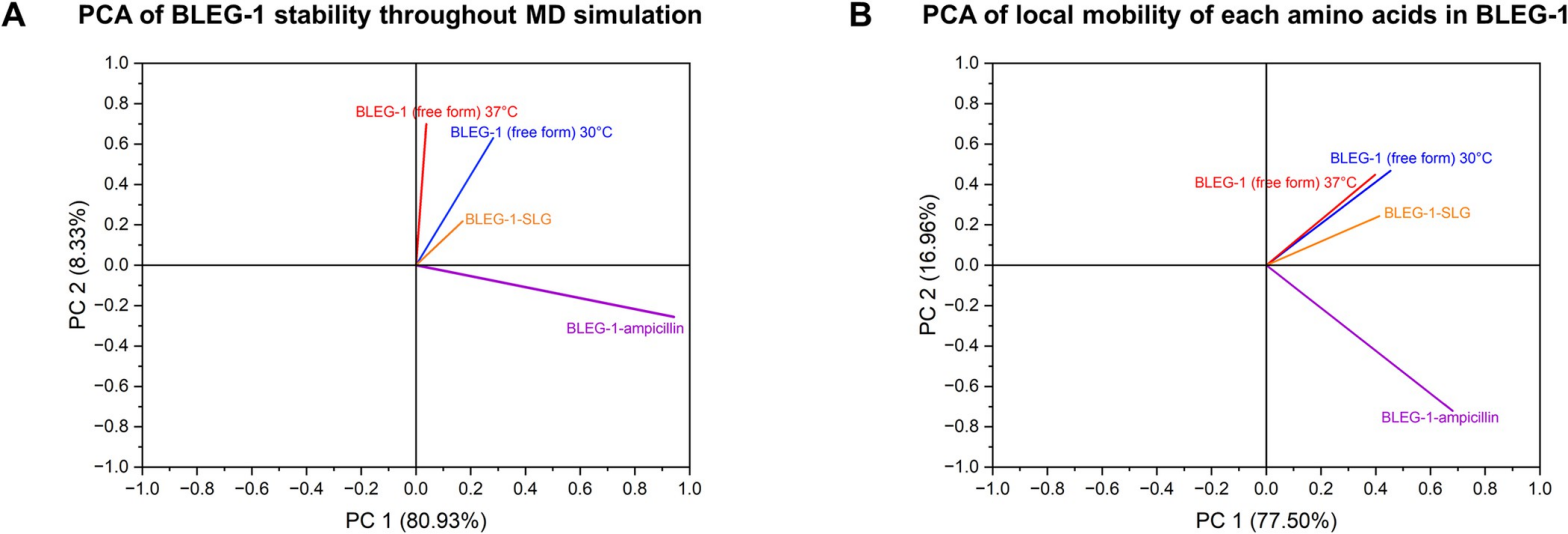
Principal component analysis (PCA) of (A) protein stability throughout MD simulation and (B) local mobility of each amino acids of BLEG-1 (free form) at both 30°C and 37°C, BLEG-1–ampicillin and BLEG-1–SLG complexes.

The correlation of protein stability throughout the MD simulation (RMSD), as well as the local mobility of residues (RMSF) of BLEG-1 (free form), BLEG-1–ampicillin and BLEG-1–SLG complexes were investigated using PCA. The PCA loading plots in Fig 10 reveal that BLEG-1–ampicillin complex demonstrated negative correlation to BLEG-1 free enzyme at both 30°C and 37°C, and BLEG-1–SLG complex, which were positively correlated on PC2 axis, suggesting relatively dissimilar protein stability and mobility of each amino acid of BLEG-1–ampicillin complex than the other three models. This is in parallel to the above mentioned postulation that the ampicillin binding induced conformational flexibility of BLEG-1, whereas BLEG-1 free enzyme and BLEG-1–SLG complex similarly inherent relatively higher stability. Although the positive correlation of BLEG-1 (free form) and BLEG-1–SLG complex suggested their resemblance, it is important to note that their protein dynamics are not identical. This is not only reflected in the differences in their respective eigenvectors, but also the RMSD and RMSF analyses described earlier.

Molecular mechanics Poisson–Boltzmann surface area (MM/PBSA) calculation was used to estimate the binding energies of dual substrates to BLEG-1 during MD simulations, in which the binding of ampicillin and SLG to the enzyme resulted in binding scores of -103.4475 kcal/mol and -260.8425 kcal/mol respectively. Although theoretically more positive scores reflect better binding and *vice versa*, the scores obtained in this case cannot be used as a yardstick to ultimately pinpoint better binding options between the two ligands. This is because fluidity in the interaction at initial binding between BLEG-1 and SLG was observed (due to substrate orientation), which may have resulted in the “weaker” binding score, as opposed to ampicillin which portrayed a more “fixed” binding mode with BLEG-1.

### Enzyme activity of BLEG-1 variants determined the substrate binding modes in BLEG-1

**Fig 11:**
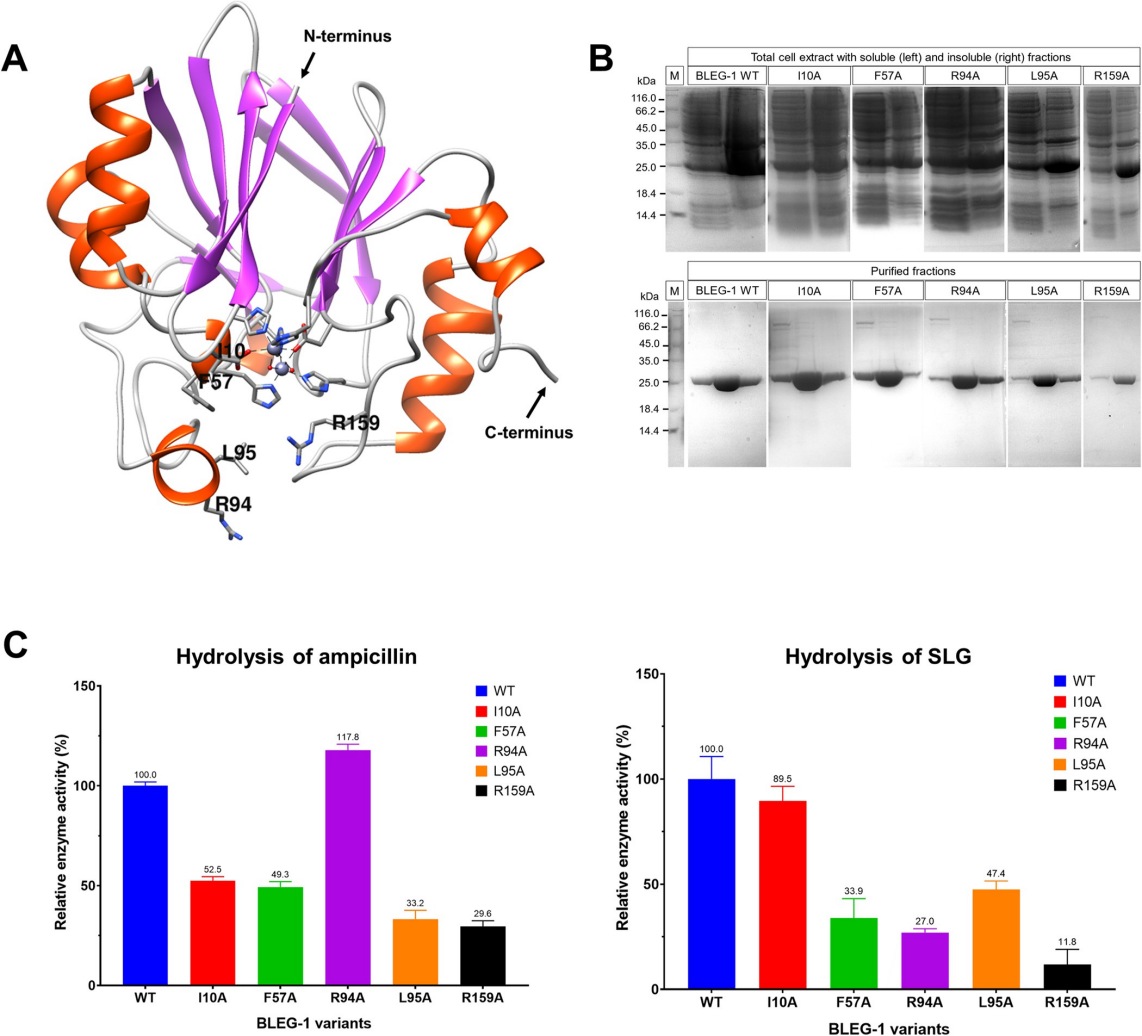
(A) Active site residues of BLEG-1 selected for site-directed mutagenesis analysis. (B) Overexpression and purification of WT and variants BLEG-1 recombinant proteins (molecular weight: 26 kDa), lane M refers to unstained protein marker (Thermo Fisher Scientific, USA) with size range of 14.4–116.0 kDa. (C) Effect of selected mutations toward the hydrolysis of ampicillin (left) and SLG (right). Relative enzyme activity refers to the ratio between the activity of variant BLEG-1 and the activity of WT BLEG-1 (control) expressed in percentage. One unit of enzyme activity is defined as the amount of enzyme catalyzed the formation of one micromole of product per minute under optimized assay conditions described in “Materials and Methods” section.

Based on the MD analyses, five residues which are most possibly significant for both ampicillin and SLG binding in BLEG-1 are Ile-10, Phe-57, Arg-94, Leu-95, and Arg-159. They were selected for alanine scanning (Fig 11A). Although His-131 and His-191 could also be important for substrate binding in BLEG-1, they were not targeted for mutagenesis as they are zinc coordinating residues. Targeting them could destabilize the metal coordination site and lead to protein misfolding [48].

WT and variants BLEG-1 recombinant proteins were overexpressed in both soluble and insoluble states in *E*. *coli* BL21(DE3), in which the soluble proteins were purified to 95% homogeneity using Ni-NTA chromatography. The elution fractions containing WT and variants BLEG-1 proteins with highest purity (i.e. in the absence of non-targeted proteins as observed in SDS-PAGE gels) were pooled for buffer exchange by dialysis. Following overnight dialysis, typically 1.5–7 mg of purified WT and variants BLEG-1 with 99% purity were obtained from 0.5-liter bacterial culture (Fig 11B).

Enzymatic assays of variants BLEG-1 toward the hydrolysis of ampicillin and SLG were conducted using the purified recombinant proteins. Their relative enzyme activities to WT BLEG-1 were calculated to determine the effect of selected mutations on substrates binding and catalytic reactions. Fig 11C (left) shows the mutated proteins i.e. I10A, F57A and L95A exhibited >50% reduction in MBL activity, indicating that Ile-10, Phe-57, and Leu-95 are crucial for interaction and hydrolytic activity toward ampicillin. These results correspond to the MD data that these hydrophobic amino acids in the N-terminal domain of BLEG-1 are involved in the interaction with the ASC of ampicillin. However, among these targeted hydrophobic residues, L95A variant showed lower MBL activity than that of I10A and F57A variants, suggesting that Leu-95 may be more important for ampicillin binding of BLEG-1. In light of the MD trajectories, positional adjustment of ampicillin caused its ASC to be projected outward from the metal center, moving the phenyl ring for about 3 Å closer to the hydrophobic side chain of Leu-95 and imposed more interactions between them. The lower MBL activity of L95A variant suggests that the final orientation of ampicillin (at 100 ns) simulated in our MD experiment could represent the actual scenario of ampicillin binding in BLEG-1. In contrast, the alanine substitution of Arg-94 yielded an enzyme that retained the MBL activity of WT, suggesting Arg-94 may not be involved in ampicillin binding as postulated in our MD experiment. Although Arg-94 and Leu-95 are adjacent to each other, low hydrophobicity of arginine as well as the projection of the guanidino group of Arg-94 to the phenyl ring of ampicillin (MD data) may hinder their interactions. In the C-terminal domain, the mutation of Arg-159 significantly reduced MBL activity (~70%) in BLEG-1. This suggests the interaction between Arg-159 and the thiazolidine ring of ampicillin by hydrogen bond observed in the MD trajectories could in fact be involved in the stabilization of ampicillin during catalysis.

On the other hand, GLXII activities of F57A, R94A, L95A, and R159A BLEG-1 variants were >50% lower than that of WT (Fig 11C, right). This correlates to the MD analysis that Phe-57, Arg-94, Leu-95 and Arg-159 are important for the accommodation of SLG in the active site. Ile-10, which could not bind SLG due to its substantial distance from the substrate did not affect GLXII activity upon alanine substitution. Leu-95 which was proposed to be involved in the conformational displacement of SLG, together with Arg-94 and Arg-159, displayed less severe reduction in GLXII activity in BLEG-1 compared to the arginine residues. This is probably because the interactions between the Glu-moiety of SLG and the polar side chain of arginine residues are stronger than the hydrophobic interactions and temporal hydrogen bond established by the isobutyl side chain and the skeletal carboxylate group of Leu-95, respectively. As proposed in the MD analysis, Phe-57 which stabilized the SLG molecule in the vicinity of zinc ligands, exhibited significant decrease in GLXII activity by approximately 70%. This provides further credence that the final binding pose of SLG in BLEG-1 predicted by MD simulation (at 100 ns) could be promising. If SLG is solely bound by the residues in the C-terminal domain of BLEG-1 as described in our previous docking study, no interactions would be observed between SLG and the residues in N-terminal domain i.e. Phe-57, Arg-94, and Leu-95.

### Proposal of catalytic mechanisms of BLEG-1

**Fig 12:**
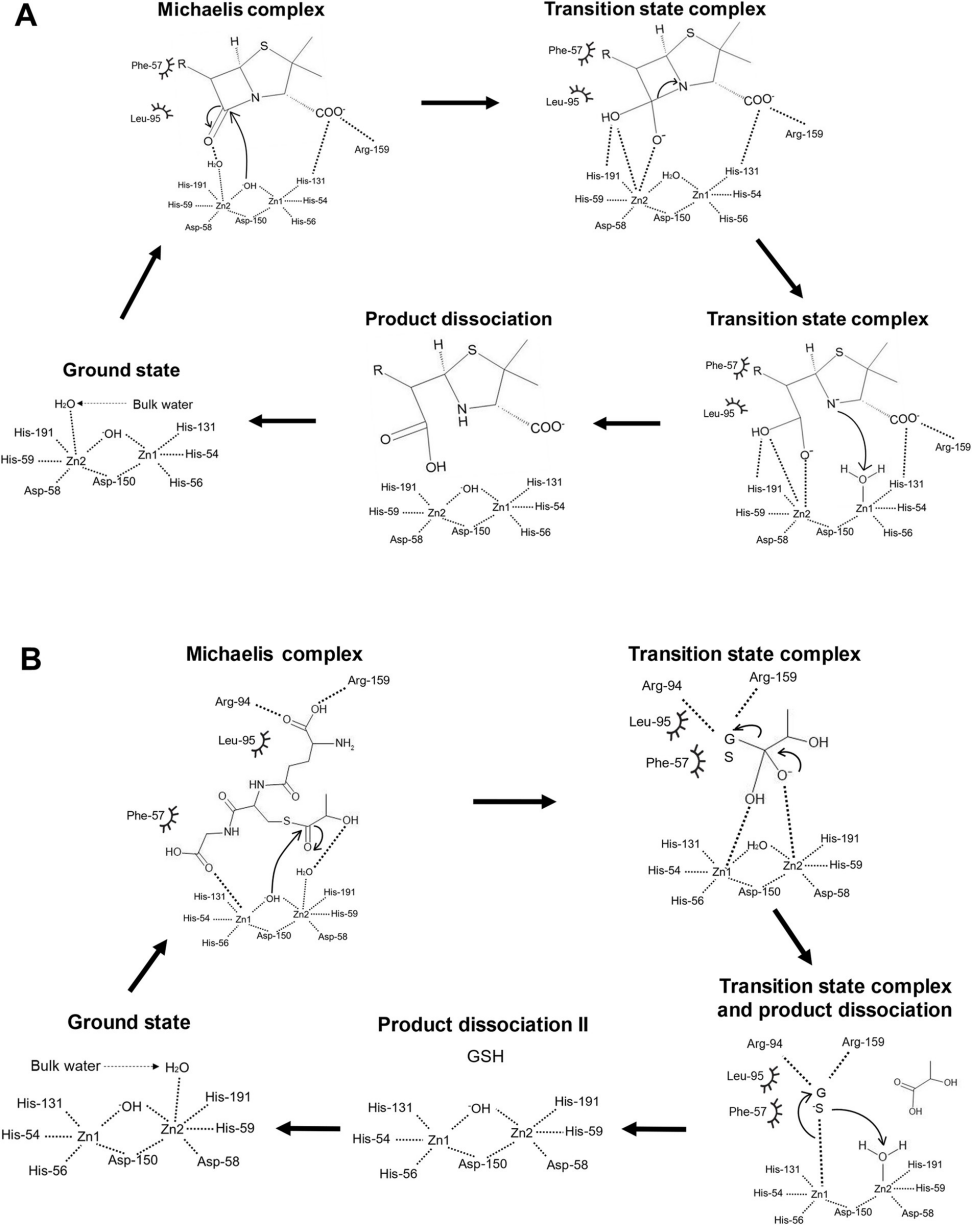
Proposed catalytic mechanisms of BLEG-1 toward the hydrolysis of (A) β-lactam antibiotics and (B) SLG. Spoked arc represents hydrophobic interaction; and dash represents hydrogen bond and/or electrostatic interaction. The chemical structures were drawn using MedChem Designer (version 5.5.0.11).

Previous research suggested that both B3 MBL and GLXII could have foster similar mechanistic reactions, due to their resemblance to binuclear metallosite where catalysis occurs [18, 46, 47]. Hence, the catalytic mechanisms of BLEG-1 toward the hydrolysis of β-lactam antibiotics and SLG could be similar and were proposed based on the results from MD simulations of WT BLEG-1 and enzymatic assays of mutated BLEG-1 variants described in previous sections (Fig 12). In line with most B3 MBLs and GLXIIs, the dizinc ions of BLEG-1 could be crucial in stabilizing the carbonyl group of β-lactam and SLG, as well as the negatively-charged reaction intermediate during catalysis [18, 49, 50]. The zinc ions could also lower the pKa value of water, making it a potent nucleophile to attack the carbonyl carbon of β-lactam and lactoyl carbon of SLG, respectively [44]. Since the bridging water (Wat1) is coordinated by two zinc cations, it has higher tendency to be deprotonated into hydroxide ion, and served as a stronger hydrolytic nucleophile than the apical water (Wat2) [18, 46] in BLEG-1.

When a β-lactam molecule binds at the active site of BLEG-1, its penam ring is stabilized by His-131, Arg-159 and the apical water of the enzyme, whereas the carbon substituent (R group) is stabilized by the hydrophobic amino acids in the N-terminal domain i.e. Phe-57 and Leu-95 (Fig 12A). It is believed that these interactions may also take part in the stabilization of transition state during the catalytic reaction. Hydrolysis of β-lactam antibiotic in BLEG-1 is most probably initiated by the attack of bridging hydroxide on the carbonyl carbon of the scissile β-lactam ring. This process forms an anionic intermediate whereby the oxyanion hole could be stabilized through (i) electrostatic interactions between the negatively charged oxygen and Zn2 ion; and (ii) interactions with the polar hydroxyl group (–OH), either by Zn2 (via electrostatic attraction) or His-191 (via hydrogen bond or dipole interactions) [2, 51]. Stabilization of the anionic intermediate will lead to the cleavage of C–N bond and cause delocalization of electrons to the nitrogen atom, resulting in an anionic nitrogen which may later deprotonate the apical water molecule [52, 53]. This will contribute to the formation of product i.e. open-ring derivative of β-lactam, and a hydroxide ion in the zinc coordination site [47]. As the product is dissociated, the metal center of BLEG-1 could be recovered by the coordination of water molecule from bulk solvent [51] (Fig 12A).

As SLG binds to BLEG-1, the coordinated zinc ions, His-191, and apical water stabilize the Cys-moiety of SLG near the dizinc center, while Phe-57 interacts with the Gly-moiety of SLG in the N-terminal region (Fig 12B). Residues on the flanking active site loops i.e. Arg-94, Leu-95, and Arg-159 further stabilize the SLG by interacting with its Glu-moiety. Once the SLG molecule is orientated for nucleophilic attack, the bridging hydroxide ion of BLEG-1 could attack the lactoyl carbonyl of SLG. This forms an anionic intermediate which may be stabilized by Zn2 and Zn1 by interacting with its alkoxide ion (RO^–^) and hydroxyl group, respectively. Such interactions facilitate C–S bond fission, which will lead to the formation of D-lactate (i.e. the first product), and a thiolate intermediate (GS^−^) that could be stabilized by Zn1 via electrostatic attractions [39, 41]. The thiolate ion will then be protonated by apical water, releasing the second product i.e. glutathione (GSH) and recover the zinc coordination site as mentioned above (Fig 12B).

## Discussion

In addition to the biochemical and structural characterizations of BLEG-1 by Tan et al. (2017) [12] and Au et al. (2021) [13], our CD results proved that the enzyme binds to ampicillin and S-D-lactoylglutathione (SLG), giving credence to our previous postulation that BLEG-1 possess an active site topology which could accommodate both substrates [12, 13]. The comparable binding affinities (K_d_) of BLEG-1 toward ampicillin and SLG are also in line with the catalytic properties, whereby BLEG-1 promiscuously catalyzed the hydrolysis of both substrates at similar magnitude [13]. Hence, the differences in the binding scores of ampicillin and SLG towards BLEG-1 as estimated by MD data may not be appropriate to be used as an absolute indicator in this case, particularly when fluidity in binding was observed between SLG and BLEG-1 due to ligand repositioning in the active site of the enzyme.

Although both ampicillin and SLG bind to the same pocket in BLEG-1, however, lower topological polar surface area (TPSA) of ampicillin (138 Å^2^) compared to SLG (221 Å^2^) (https://pubchem.ncbi.nlm.nih.gov, accessed on Jan 5^th^, 2023), suggests differences in the enzyme-substrate interactions as well as the influences on protein dynamics and stability. Our MD data suggested BLEG-1 interacted with ampicillin mainly by hydrophobic interactions, thus promoted structural flexibility in the enzyme and have led to destabilization of ampicillin complexed structure. The binding of SLG, in contrast, was predominantly mediated by hydrogen bonds in BLEG-1, thereby contributed to greater stabilization effect to the enzyme structure. This was reflected in the lower RMSD value of the overall structure, as well as decreased fluctuation (RMSF) of most flexible zones in BLEG-1 upon SLG binding.

Further investigation of the dynamic behavior of BLEG-1 and its substrate complexes revealed correlation between structural dynamicity and the binding of dual substrates in the enzyme. The overall structure of BLEG-1 is stabilized by a rigid core that is comprised of β-sheets and coordinated zinc ions via extended hydrogen bond networks, whereas the loops and α-helices on protein surfaces exhibited dynamicity in explicit solvent. Flexibility of the active site loops i.e. loop7+α3+loop8, loop12 and loop14 are prominent in facilitating the binding, orientation and stabilization of both ampicillin and SLG in BLEG-1. Loop motions determine the adjustment to the conformation of substrate molecules, in which their scissile carbonyl group are more accessible to nucleophilic attack at the zinc coordination site of BLEG-1. The significance of loop motions for the binding of β-lactam antibiotics in BLEG-1 is similar to most B3 MBLs, such as L1, GOB, and FEZ-1 [42, 44, 54]. In the case of L1 B3 MBL, stopped-flow fluorescence and MD simulations analyses have provided ample evidence that the “flapping” active site loops i.e. loop9 and loop11 (also known as α3-β7 loop and β12-α5 loop, respectively) (S2 Fig) are mobile and involved in substrate binding [55, 56]. Despite the shorter loop length of loop7+α3+loop8 and loop12 in BLEG-1, these two loops could behaved flexibly as loop9 and loop11 in L1, explaining the biochemical similarities between BLEG-1 and B3 MBLs against a broad range of β-lactam antibiotics [12]. In contrast to B3 MBL, long and flexible loops are absent in most GLXIls, the binding of SLG was observed at their wide and shallow active site cleft, with the association of a small α-helical domain at C-terminus [20, 46, 57]. Due to the absence of α-helical domain in BLEG-1, initial binding of SLG in its C-terminal region was not stable and have led to subsequent substrate displacement, which could be mediated and stabilized by the interactions between fluctuating amino acids and the Glu-moiety of SLG at the “entrance” of the binding pocket. This was justified by the notable reduction of GLXII activity in variants BLEG-1 with alanine substituted for Arg-94, Leu-95, and Arg-159, in addition to the decrease in RMSF values of these three residues in the MD data of BLEG-1–SLG complex. Although Cameron et al. (1999) suggested that the binding of Glu-moiety of SLG may not be necessary in GLXII, it seems to be important for GLXII which lacks the C-terminal α-helical domain; with the criterion that the protein possess amino acids with flexible side chains on the active site structures.

Similar to most B3 MBLs such as L1, FEZ-1, and BJP-1, the active site groove of BLEG-1 in the N-terminal domain is predominantly composed of hydrophobic amino acids which could be essential for the recruitment and stabilization of β-lactam substrates with C5/C6 substituents [43–45]. According to the mutagenesis studies on L1 MBL by Simm et al. (2002) and Carenbauer et al. (2002), alanine substitution of hydrophobic amino acids positioned in the N-terminal extension i.e. Trp-17, and the gorge-forming active site loop at N-terminus (loop9 or α3-β7 loop) i.e. Ile-164 and Phe-158 (BBL standard numbering), yielded mutant proteins with significantly lower binding affinities toward β-lactam antibiotics [58, 59]. The mutation of Ile-10 and Leu-95 of BLEG-1, which located in similar vicinity as Trp-17, Ile-164 and Phe-158 of L1, decreased the MBL activity of BLEG-1, thus supporting the proposed role of Ile-10 and Leu-95 in ampicillin binding. Notably, the large loss in MBL activity of L95A variant inferred the possibility that Leu-95 could be stabilizing the ASC of ampicillin which projected outward the catalytic core and exposed to solvent, in actual scenario. This showed resemblance to the binding mode of penicillin in L1 MBL (PDB ID: 6U0Z), whereby the phenyl group of penicillin could have stabilized by Phe-145 (also denoted as Phe-158 following the BBL numbering scheme) in L1 MBL [42]. In other B3 MBLs, amino acids with hydrophobic side chain were also conserved at this position and were orientated toward the metal center as Leu-95 of BLEG-1 and Phe-158 of L1 MBL. They are Met-138 of GOB-18 (PDB ID: 5K0W) and Tyr-156 of FEZ-1 (PDB ID: 1JT1) (S4 Fig), suggesting the roles of these hydrophobic residues in stabilizing the aromatic substituent of β-lactam antibiotics. Beyond the active site loops, the implications of aromatic residues neighboring the metal coordination site of B3 MBL for β-lactam binding have not been delineated. Our MD simulation speculated the potential of Phe-57 in forming stacking interactions with the phenyl group of ampicillin. Such interaction could be favorable in stabilizing β-lactams with bulky substituents during catalysis. Interestingly, amino acids with phenyl ring are structurally conserved at the same position in some B3 MBLs, such as AIM-1 (Phe-119; PDB ID: 4AWY), FEZ-1 (Phe-119; PDB ID: 1JT1) and GOB-18 (Tyr-101; PDB ID: 5K0W). However, the absence of crystallographic structures for the enzyme-substrate complexes of these B3 MBLs hampers the understanding of the role of Phe/Tyr in β-lactam binding. Currently available structures which unveiled substrate interactions of B3 MBLs mostly involve L1 and SMB-1, for example, L1–penicillin (PDB ID: 6U0Z), L1–meropenem (PDB ID: 6UAH), L1–imipenem (PDB ID: 6UAF), L1–moxalactam (PDB ID: 6U13), L1–faropenem (PDB ID: 7A63), L1–ertapenem (PDB ID: 7O0O), and SMB-1–imipenem (PDB ID: 5B1U) [42, 60]. Structural superimpositions revealed that Phe-57 in BLEG-1 correlates to alanine and glutamic acid in L1 and SMB-1 respectively. However, there were no interactions observed between the Ala/Glu residues and β-lactam molecule in the enzyme-substrate complexes of L1 and SMB-1. In our study, removing the aromatic side chain of Phe-57 resulted modest reduction of MBL activity in BLEG-1. Therefore, though the amino acid of high hydrophobicity at this position may not be compulsory for ampicillin binding, it apparently played a role during catalysis in BLEG-1. Another key interaction possessed by BLEG-1 on ampicillin molecule is the hydrogen bond between Arg-159 and the thiazolidine ring of β-lactam. This interaction was also predicted in L1 MBL, which seemed to be mediated by a serine residue at position 187 [43]. The superimposed structure of BLEG-1 and L1 demonstrated that these two residues are located at similar zone in respective protein structures. Although substitution of Ser-187 did not affect much of L1 enzymatic activity, the decrease in intermediate formation detected via stopped–flow UV-Visible spectrophotometric analysis inferred the involvement of this residue in substrate stabilization [59]. In the case of BLEG-1, alanine substitution of Arg-159 resulted less active enzyme against ampicillin. Hence, the participation of Arg-159 in the hydrolysis of ampicillin is without a doubt.

Unlike B3 MBLs, the interaction of SLG in GLXII remains elusive till present time, especially for bacterial GLXII. Thus, it is difficult to assign the relevance of amino acid residues in SLG binding in BLEG-1, as well as other GLXIIs. However, given that substrate binding residues of GLXIIs are generally conserved among prokaryotic, fungal, yeast, and plant GLXIIs [20], detailed understanding of SLG binding in BLEG-1 was attempted by referring to the reports on both prokaryotic and eukaryotic GLXIIs. Based on available crystal structures of common GLXII e.g. human GLXII (hGLXII) (PDB ID: 1QH5), *Salmonella typhimurium* GloB (PDB ID: 2QED), *Arabidopsis thaliana* GLXII (PDB ID: 1XM8; 2Q42) in addition to the report by Cameron et al. (1999) [46], substrate interaction of GLXII seems to be more dependent on polar and/or charged amino acids, due to the prevalence of these residues in the active site cavities of these enzymes. In contrast, despite the analogous packing of C-terminal active site loop in all GLXIIs, YcbL GLXII-2 (an unusual GLXII) and BLEG-1 (in this study), which both lack of an α-helical domain at C-terminus, possessed an active site groove of higher hydrophobicity in their N-terminal regions [13]. Therefore, different binding modes of SLG in BLEG-1 and common GLXIIs can be expected. Based on the complexed structure of hGLXII, the Gly-moiety of HBPC-GSH (a substrate analogue of SLG) is interacted by Arg-249 and Lys-252 positioned in the C-terminal α-helical domain of the protein [46]. The role of arginine residue at this position for the binding of SLG is further inferred by the significant loss of GLXII activity in *A*. *thaliana* GLXII after substitution of Arg-248 (corresponds to Arg-249 in hGLXII) to tryptophan. For the absence of α-helical domain as in the case of BLEG-1, we suggest the Gly-moiety of SLG is accommodated by Phe-57 at the N-terminus of BLEG-1. This is implied from the decrease of GLXII activity when Phe-57 was replaced by alanine, in addition to the interactions observed between Phe-57 and the Gly-moiety of SLG in the MD trajectories. For the interaction with Glu-moiety of SLG, though it was proposed to be insignificant, hGLXII–HBPC-GSH complexed structure showed that Lys-143 forms hydrogen bond with the carbonyl oxygen of this substrate substructure. The importance of this lysine residue was further confirmed in the mutagenesis study by Zang et al. (2001) [47], whereby alanine substitution of Lys-142 in *A*. *thaliana* GLXII (corresponds to Lys-143 in hGLXII) produced mutant protein with modest reduction in enzyme activity, suggesting weak binding of SLG in *A*. *thaliana* GLXII. In bacterial glyoxalase II, the corresponding lysine is substituted by arginine i.e. Arg-136 in GloB (PDB ID: 2QED), Arg-160 in YcbL (PDB ID: 2XF4), and Arg-159 in BLEG-1. Based on the side chain projection of this arginine in GloB and YcbL, Suttisansanee & Honek (2011) [23] speculated that Arg-136 in GloB may not be able to interact with the Glu-moiety of SLG, but this may not be the case in YcbL [23]. This is because the guanidino side chain of Arg-136 in GloB forms hydrogen bond with Asp-249 in the C-terminal α-helical domain, protruding the side chain away from the substrate binding cleft (S5 Fig). However, for YcbL and BLEG-1, both lacking the α-helical domain, their arginine side chain orientated toward the metal center, thereby making them probable to be involved in SLG binding. Our MD data support the proposition, whereby Arg-159 bound to the Glu-moiety of SLG and contributed to the stabilization of SLG in the active site pocket of BLEG-1. Hence, by replacing Arg-159 with alanine, significant decrease in GLXII activity was detected in BLEG-1.

In a nutshell, dynamic ensembles of BLEG-1 and its substrate complexes enabled us to resolve structural flexibility and the binding modes of promiscuous substrates to a bifunctional BLEG-1 B3 MBL. Despite the resemblance of active site topology of BLEG-1 to B3 MBL and GLXII, plasticity and amino acid properties of the substrate binding pocket could be the key factors for BLEG-1 in accommodating both β-lactam antibiotics and SLG. BLEG-1 possess flexible loops which may induce structural changes of the binding groove thereby adjusting the orientation of substrate molecules to be duly catalyzed or hydrolyzed. The hydrophobic character of the N-terminal region of BLEG-1 could be particularly crucial for BLEG-1 to accommodate β-lactam with aromatic substituents, as well as stabilizing the Gly-moiety of SLG in the absence of α-helical domain at C-terminus. Polar and charged amino acids which are distributed over the active site loops, on the other hand, play pivotal role in forming hydrogen bonds with the polar atoms of SLG, as well as the penam ring of ampicillin in BLEG-1. The characterization of site-directed BLEG-1 variants has provided important information about the involvement of key residues predicted to be significant for substrate interactions and hydrolytic activities of BLEG-1, laying the foundation for the proposition of BLEG-1 catalytic mechanisms toward ampicillin and SLG respectively.

As BLEG-1 demonstrated sequence, biochemical, structural, and mechanistic resemblance to both B3 MBL and GLXII, it could serve as a good target for drug design related to antibiotic resistance and D-lactate toxicity. Against the backdrop of combating antibiotic resistant (AR) among bacterial pathogens, many efforts have been channeled into the search for new antibiotics and therapies. In addition to this, the design and development of β-lactamase inhibitors have become equally important in the pursuit against AR, particularly after the global outbreak of NDM-1 B1 MBL in 2008 [61]. Most of clinically available inhibitors are targeted towards serine-β-lactamases (SBLs) which are constituted of β-lactamases from class A, C, and D. To date, there is still no clinically useful MBL inhibitors [62]. Graver still, SBL inhibitors that are widely available are not effective against any MBLs [63]. This certainly necessitates the urgent search of novel inhibitors against MBLs of all subclasses.

On the periphery and somewhat related to AR, it has been well documented that gut microbiotas could overproduce D-lactate in individuals who suffered from short-bowel syndrome or have undergone jejunoileal bypass surgery, causing D-lactate toxicity (acidosis). Under normal circumstances, D-lactate is produced in nanomolar concentration, through the glyoxalase system present in most microbial flora in human intestines [64]. The glyoxalase system works by converting the lethal methylglyoxal (MG) which is derived from the metabolisms of carbohydrates, lipids, and amino acids, to the non-toxic D-lactate in the presence of GSH. The process begins with the non-enzymatic reaction between MG and GSH, which forms hemithioacetal (HTA) that is recognized by glyoxalase I (GLXI) of the glyoxalase system. GLXI catalyzes the conversion of HTA to SLG, which is subsequently broken down into D-lactate and GSH catalyzed by the action of GLXII (S6 Fig) [64].

As D-lactate is not well metabolized in the human body, its overproduction and accumulation cause D-lactate toxicity, which could lead to lactic acidosis, inflammation, and encephalopathy [65, 66]. The current treatment for D-lactate toxicity usually involve the use of antibiotics [62]. However, the emergence of AR strains among the intestinal bacteria renders the antibiotics treatment ineffective [67]. It is also important to note that the consumption of antibiotics is probable in giving rise to the emergence of AR strains among the gut microbiome [68]. Until recently, some researchers hypothesized that glyoxalases inhibitors could possess bactericidal activity, thus showing their feasible application in treating AR pathogen infections [23, 69]. Although the possibility of using glyoxalases inhibitors against bacteria has not been extensively explored, several *in vitro* studies revealed the usage of novel GSH-derived inhibitors decreased the enzymatic activity of bacterial glyoxalases [70] and induced cell suicide in pathogenic bacteria [71].

In this regard, for an enzyme such as BLEG-1 that possesses both B3 MBL and GLXII activities, we believed that the presented research findings would be able to shed light on the design and development of potential inhibitors against B3 MBL, GLXII, as well as other structural homologs of BLEG-1 to help address the issues of AR and D-lactate toxicity. In future, it is of interest to probe the loop dynamicity of BLEG-1 via stopped-flow fluorescence, in addition to the structure determination of BLEG-1–substrate complexes using X-ray crystallography, to gain better insights into the structural and mechanistic features of BLEG-1, which could also be constructive to fill the current knowledge gap in MBL and GLXII research.

## Supporting information

S1 Fig Overlayed nano-ITC profiles of water and (A) BLEG-1–SLG (1:10 ratio); (B) BLEG-1–SLG (1:20 ratio); (C) BLEG-1–SLG (1:50 ratio). Nano-ITC experiments of BLEG-1 and SLG were carried out using the methods described by Selvaraju et al. (2020) [39]. The heat profile of BLEG-1 fluctuated as SLG was titrated to the protein solution, indicating possible heat cancelation by products formation during catalysis. Water-to-water injections which resulted constant peak size at heat rate of below 0.2 μJ/s indicated the nano-ITC equipment was clean before executing experiments involving BLEG-1 and SLG.
(TIF)

S2 Fig Crystal structure of L1 B3 MBL–penicillin complex (left) and a close-up of the penicillin binding site (right) (PDB ID: 6U0Z). Penicillin molecule is bound at the center of binding pocket of L1 MBL, in which the aromatic side chain and penam ring of penicillin were bound by the N-and C-terminal active site loops, respectively. The aromatic substituent of penicillin was not well defined in the crystal structure as it was proposed that the phenyl ring of penicillin was most probably oriented upward and exposed to the solvent, thus leading to such disorder during structure determination [42].
(TIF)

S3 Fig Crystal structure of human GLXII–HBPC-GSH complex (left) and a close-up of the HBPC-GSH binding site (right) (PDB ID: 1QH5). The HBPC-GSH molecule is bound in the active site pocket of human GLXII near the C-terminal region, in which its Cys-moiety is bound near the dizinc center, Gly-moiety is bound by the α-helical domain, and Glu-moiety is projected outward the catalytic core [46].
(TIF)

S4 Fig Superimposed structure of BLEG-1 and the following B3 MBLs: L1–penicillin complex (PDB ID: 6U0Z), GOB-18 (PDB ID: 5K0W), and FEZ-1 (PDB ID: 1JT1) (left). A close-up of the enzyme active sites (right) shows the side chain projection of hydrophobic amino acids in this area toward the metal coordination site.
(TIF)

S5 Fig Superimposed structure of BLEG-1 and the following bacterial GLXIIs: YcbL (PDB ID: 2XF4), and GloB (PDB ID: 2QED). A close-up of the C-terminal active site loops (right) shows the side chain projection of a structurally aligned arginine residue. Arg-136 of GloB interacts with Asp-249 in the α-helical domain, causing its side chain “swung away” from the active site pocket. In contrast, the side chain of Arg-159 of BLEG-1 and Arg-160 of YcbL projected toward the catalytic site and could behave flexibly.
(TIF)

S6 Fig The detoxification pathway of methyglyoxal (MG) through glyoxalase system [64]. (TIF)

S7 Fig (TIF)

S1 Raw images (PDF)
