# Supplementary figures and images for: Probing the substrate binding modes and catalytic mechanisms of BLEG-1, a promiscuous B3 metallo-β-lactamase with glyoxalase II properties
Source: PLoS One. 2023 Sep 6;18(9):e0291012. doi: 10.1371/journal.pone.0291012 (PMC10482274; doi:10.1371/journal.pone.0291012)

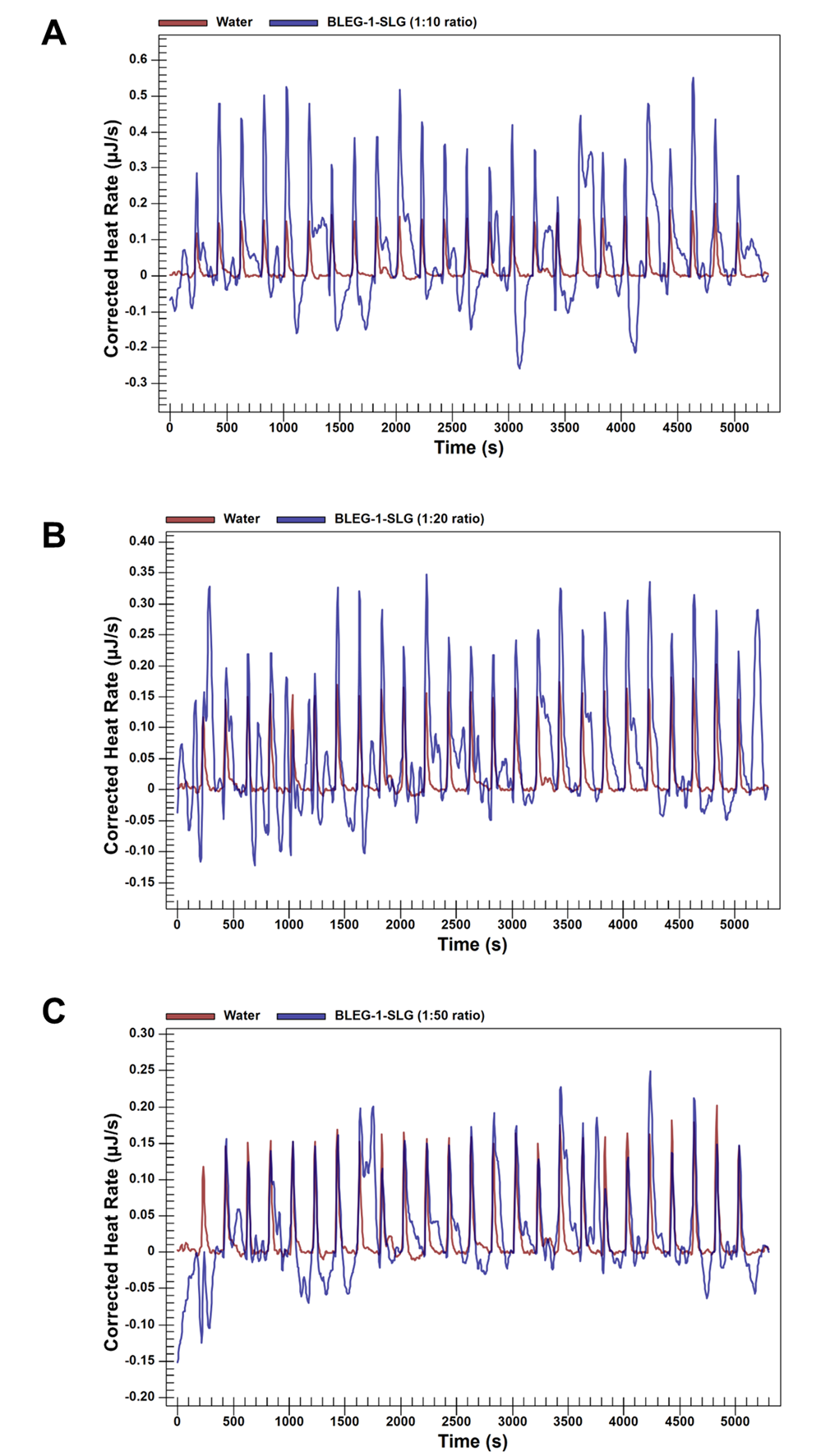

Supplement: S1 Fig — Overlayed nano-ITC profiles of water and (A) BLEG-1–SLG (1:10 ratio); (B) BLEG-1–SLG (1:20 ratio); (C) BLEG-1–SLG (1:50 ratio). Nano-ITC experiments of BLEG-1 and SLG were carried out using the methods described by Selvaraju et al. (2020) [39]. The heat profile of BLEG-1 fluctuated as SLG was titrated to the protein solution, indicating possible heat cancelation by products formation during catalysis. Water-to-water injections which resulted constant peak size at heat rate of below 0.2 μJ/s indicated the nano-ITC equipment was clean before executing experiments involving BLEG-1 and SLG. (TIF) [file pone.0291012.s001.tif]

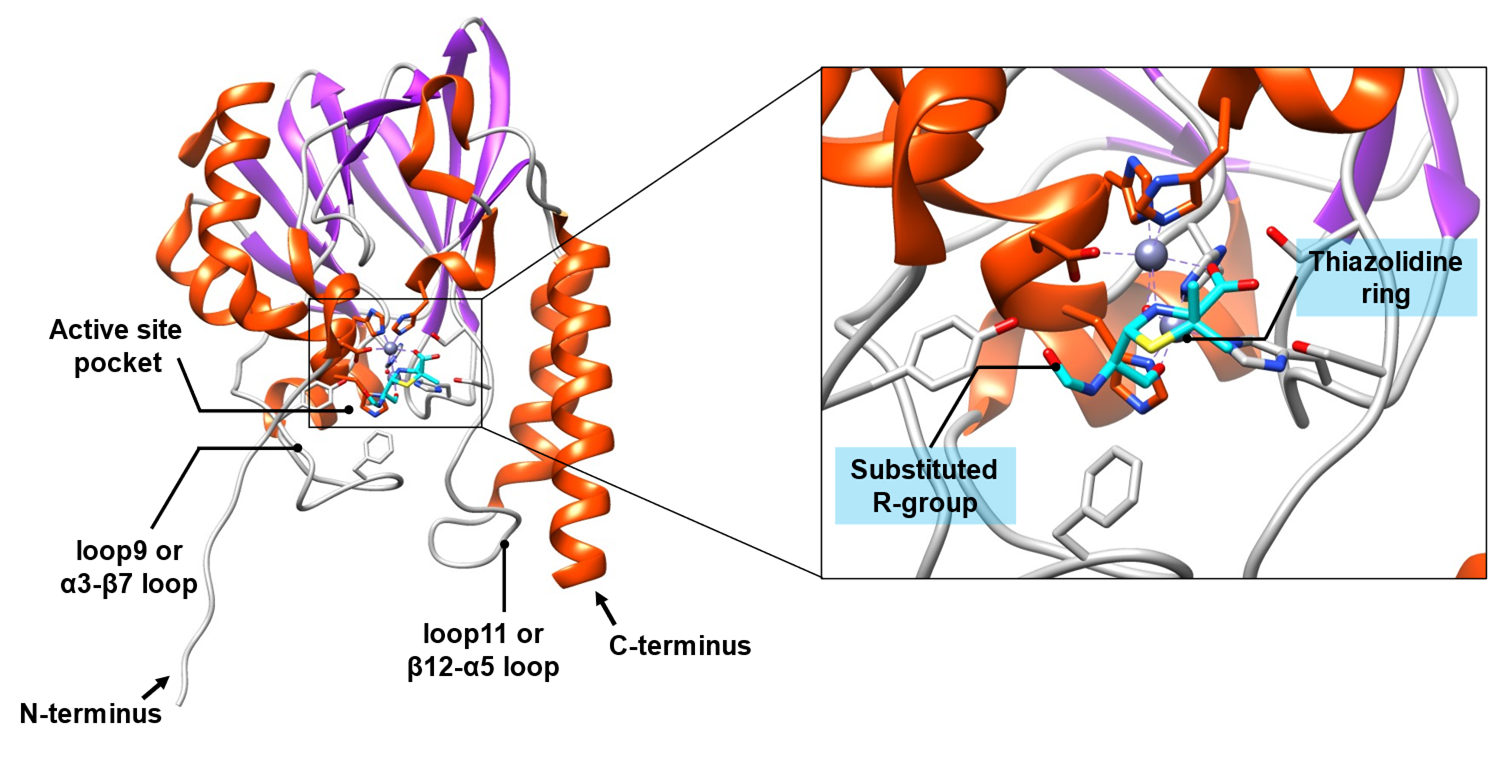

Supplement: S2 Fig — Crystal structure of L1 B3 MBL–penicillin complex (left) and a close-up of the penicillin binding site (right) (PDB ID: 6U0Z). Penicillin molecule is bound at the center of binding pocket of L1 MBL, in which the aromatic side chain and penam ring of penicillin were bound by the N-and C-terminal active site loops, respectively. The aromatic substituent of penicillin was not well defined in the crystal structure as it was proposed that the phenyl ring of penicillin was most probably oriented upward and exposed to the solvent, thus leading to such disorder during structure determination [42]. (TIF) [file pone.0291012.s002.tif]

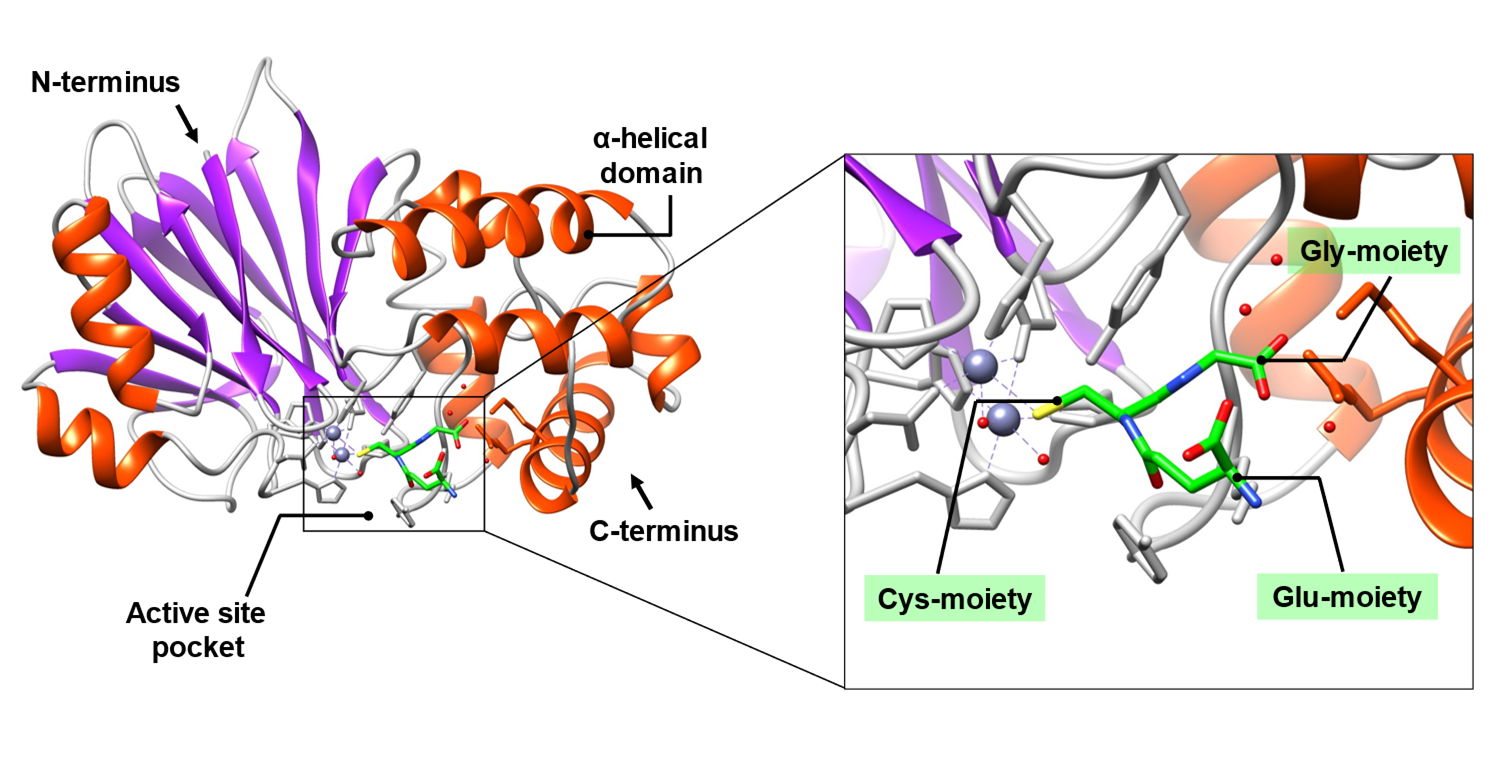

Supplement: S3 Fig — Crystal structure of human GLXII–HBPC-GSH complex (left) and a close-up of the HBPC-GSH binding site (right) (PDB ID: 1QH5). The HBPC-GSH molecule is bound in the active site pocket of human GLXII near the C-terminal region, in which its Cys-moiety is bound near the dizinc center, Gly-moiety is bound by the α-helical domain, and Glu-moiety is projected outward the catalytic core [46]. (TIF) [file pone.0291012.s003.tif]

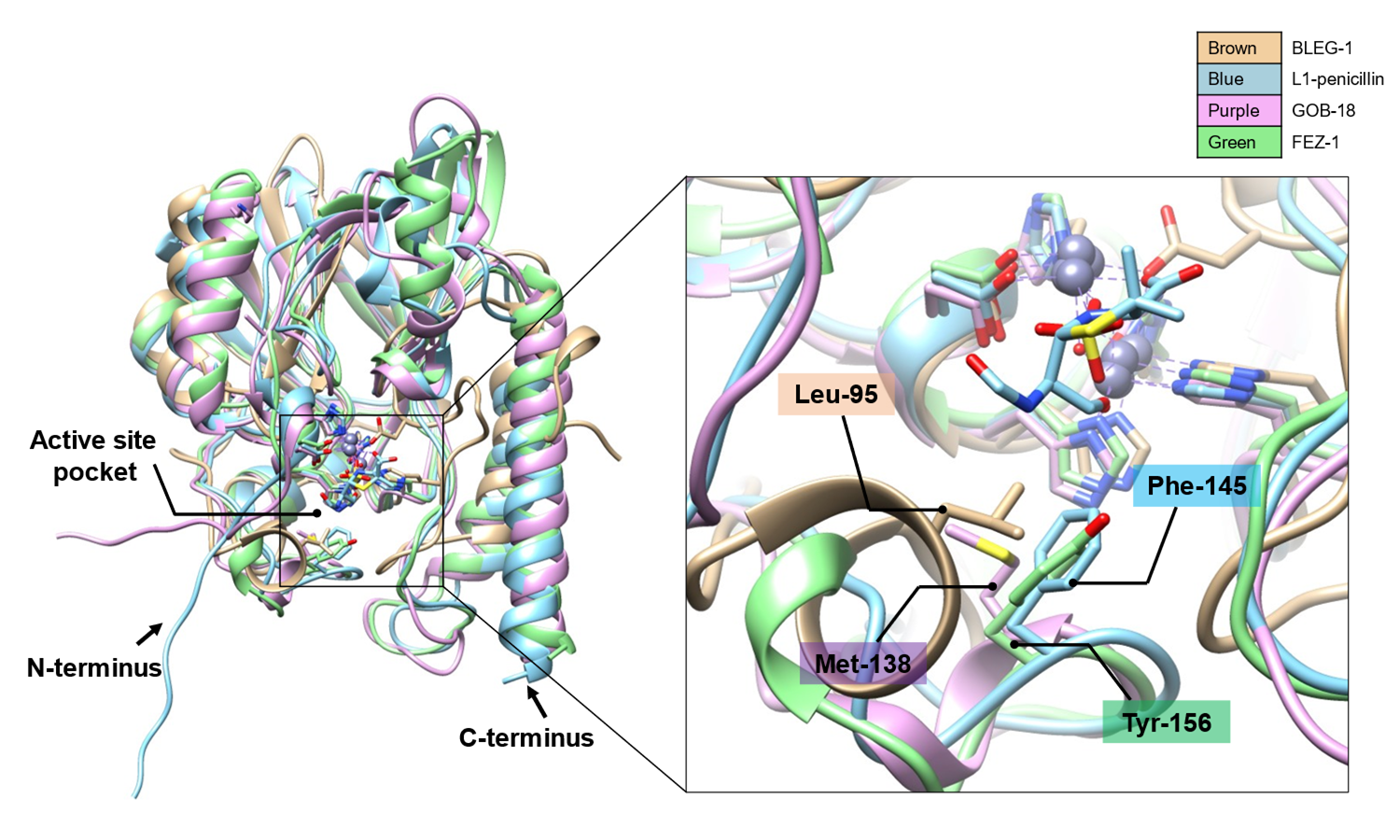

Supplement: S4 Fig — Superimposed structure of BLEG-1 and the following B3 MBLs: L1–penicillin complex (PDB ID: 6U0Z), GOB-18 (PDB ID: 5K0W), and FEZ-1 (PDB ID: 1JT1) (left). A close-up of the enzyme active sites (right) shows the side chain projection of hydrophobic amino acids in this area toward the metal coordination site. (TIF) [file pone.0291012.s004.tif]

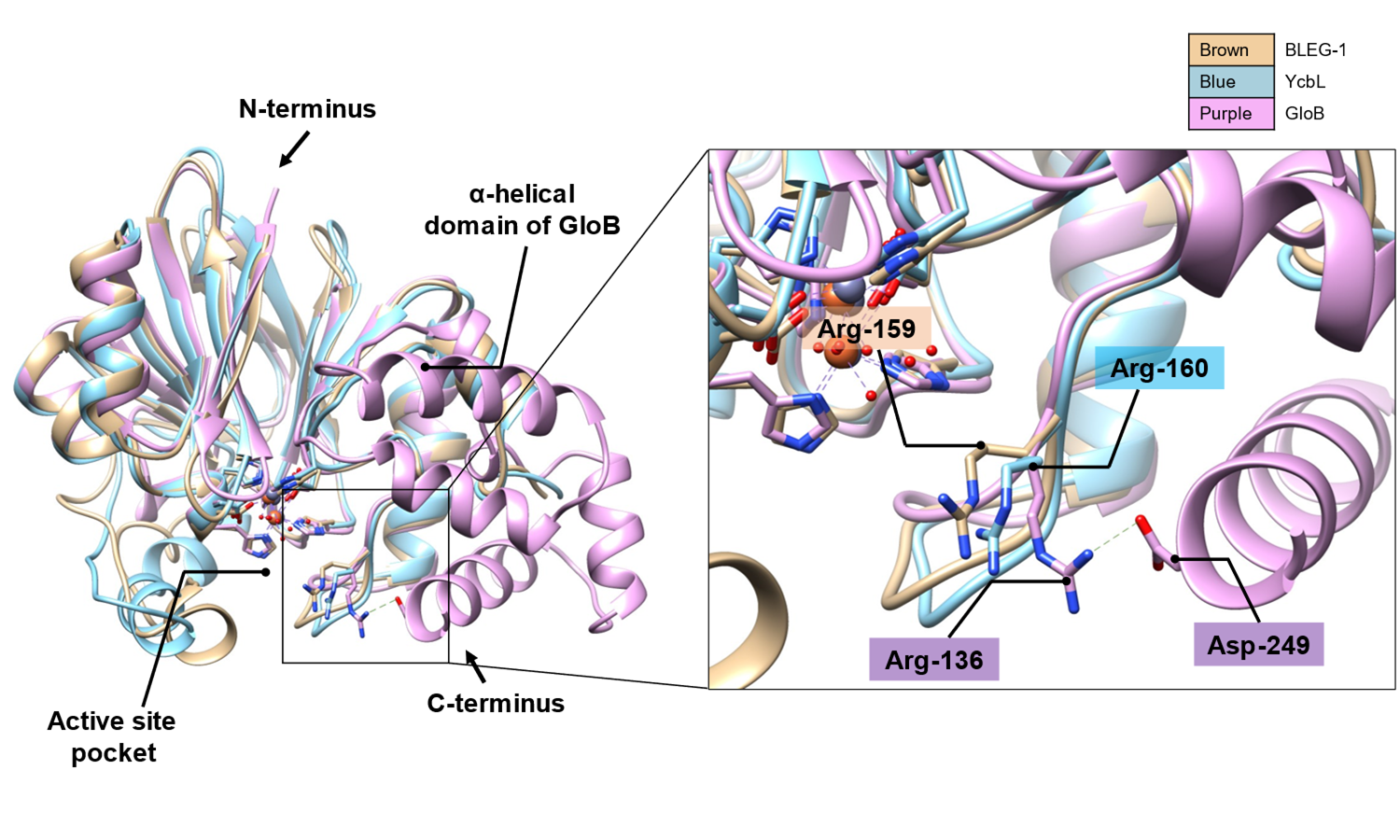

Supplement: S5 Fig — A close-up of the C-terminal active site loops (right) shows the side chain projection of a structurally aligned arginine residue. Arg-136 of GloB interacts with Asp-249 in the α-helical domain, causing its side chain “swung away” from the active site pocket. In contrast, the side chain of Arg-159 of BLEG-1 and Arg-160 of YcbL projected toward the catalytic site and could behave flexibly. (TIF) [file pone.0291012.s005.tif]

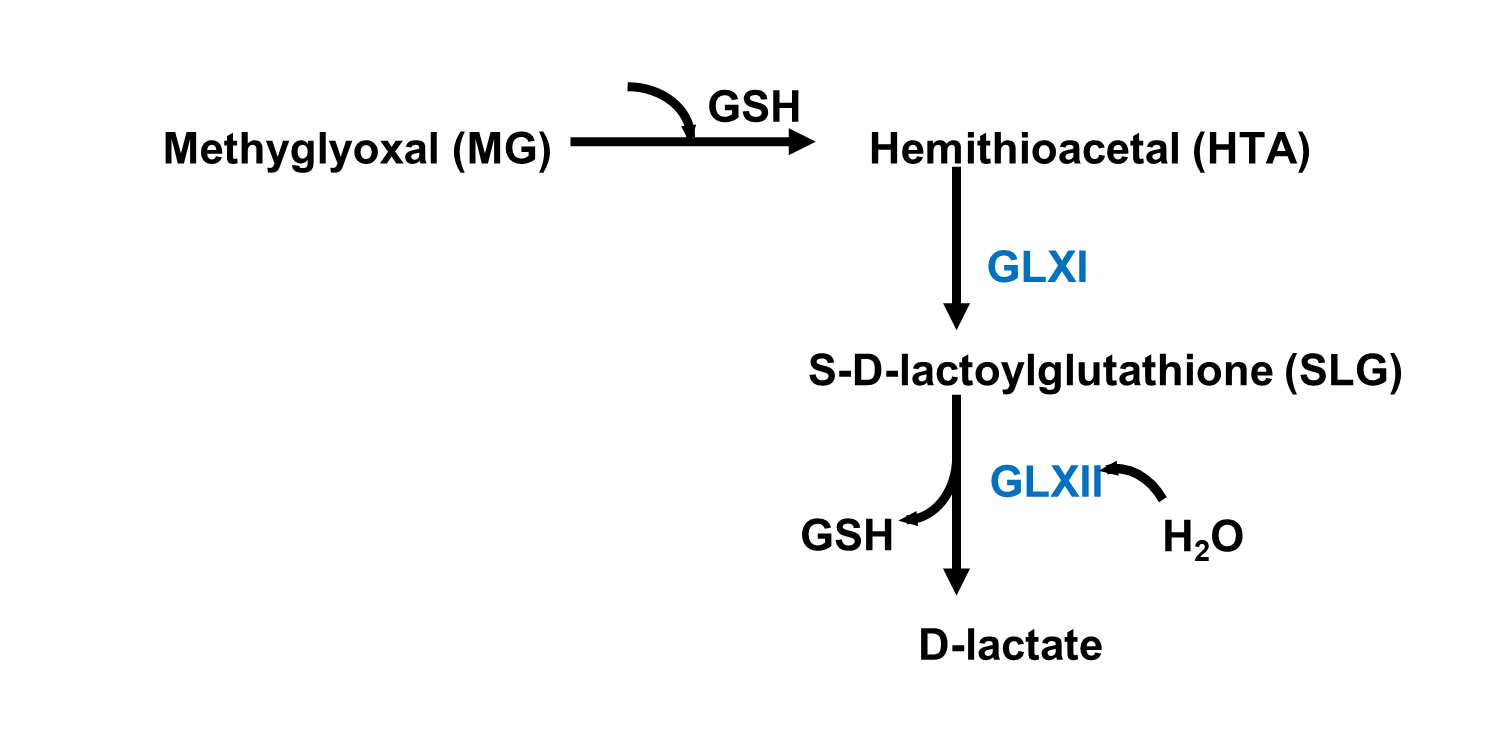

Supplement: S6 Fig — (TIF) [file pone.0291012.s006.tif]

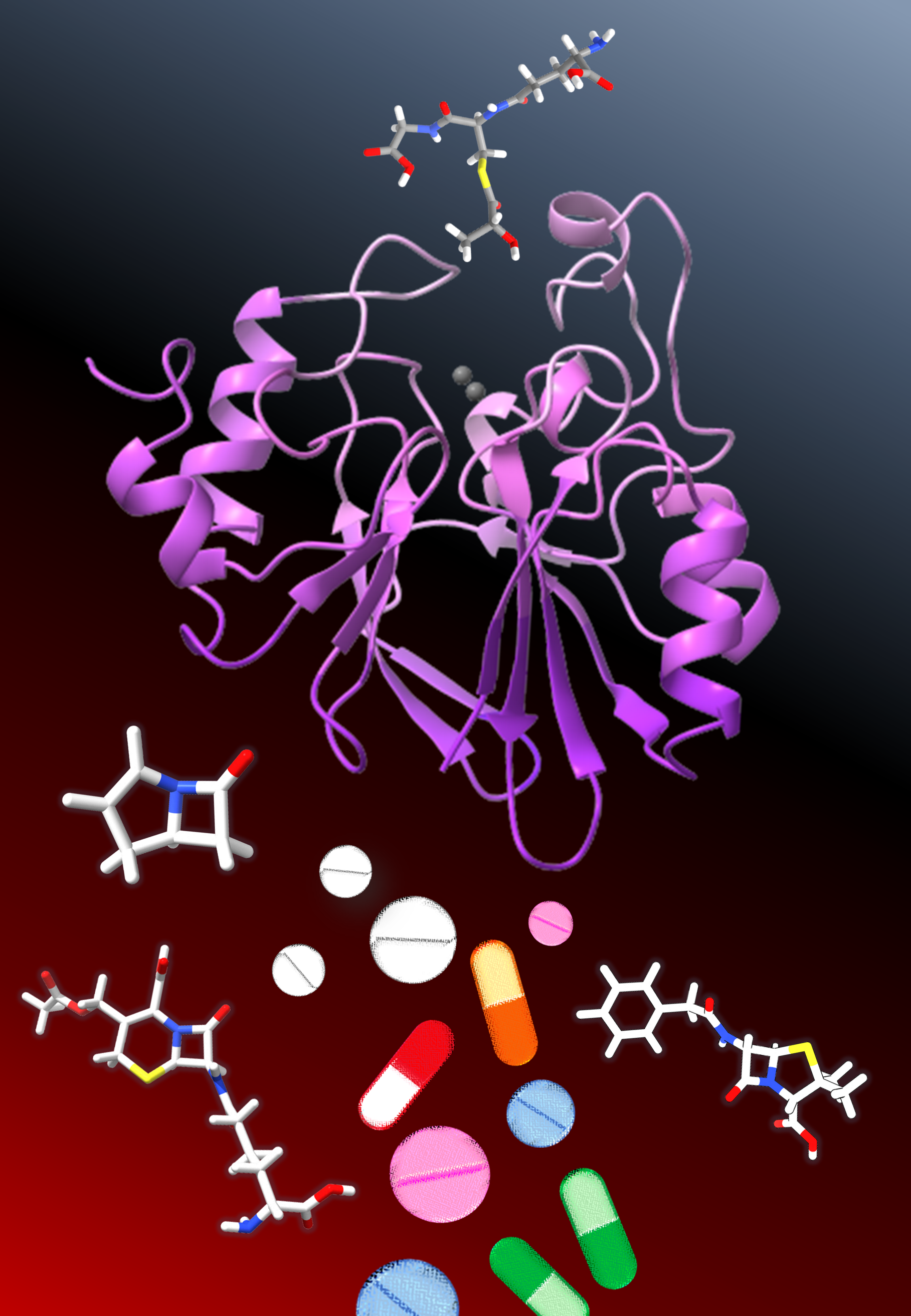

Supplement: S7 Fig — (TIF) [file pone.0291012.s007.tif]
