# Supplementary material for: Probing the substrate binding modes and catalytic mechanisms of BLEG-1, a promiscuous B3 metallo-β-lactamase with glyoxalase II properties
Source: PLoS One. 2023 Sep 6;18(9):e0291012. doi: 10.1371/journal.pone.0291012 (PMC10482274; doi:10.1371/journal.pone.0291012)
Supplement: S1 Raw images — (PDF) [file pone.0291012.s008.pdf]

This is a compilation of original gel images presented in the manuscript, titled:

Probing the substrate binding modes and catalytic mechanisms of BLEG-1, a promiscuous B3 metallo-β-lactamase with glyoxalase II properties

authored by Shaw Xian Au, Azyyati Mohd Padzil, Noor Dina Muhd Noor, Hiroyoshi Matsumura, Raja Noor Zaliha Raja Abdul Rahman and Yahaya M. Normi

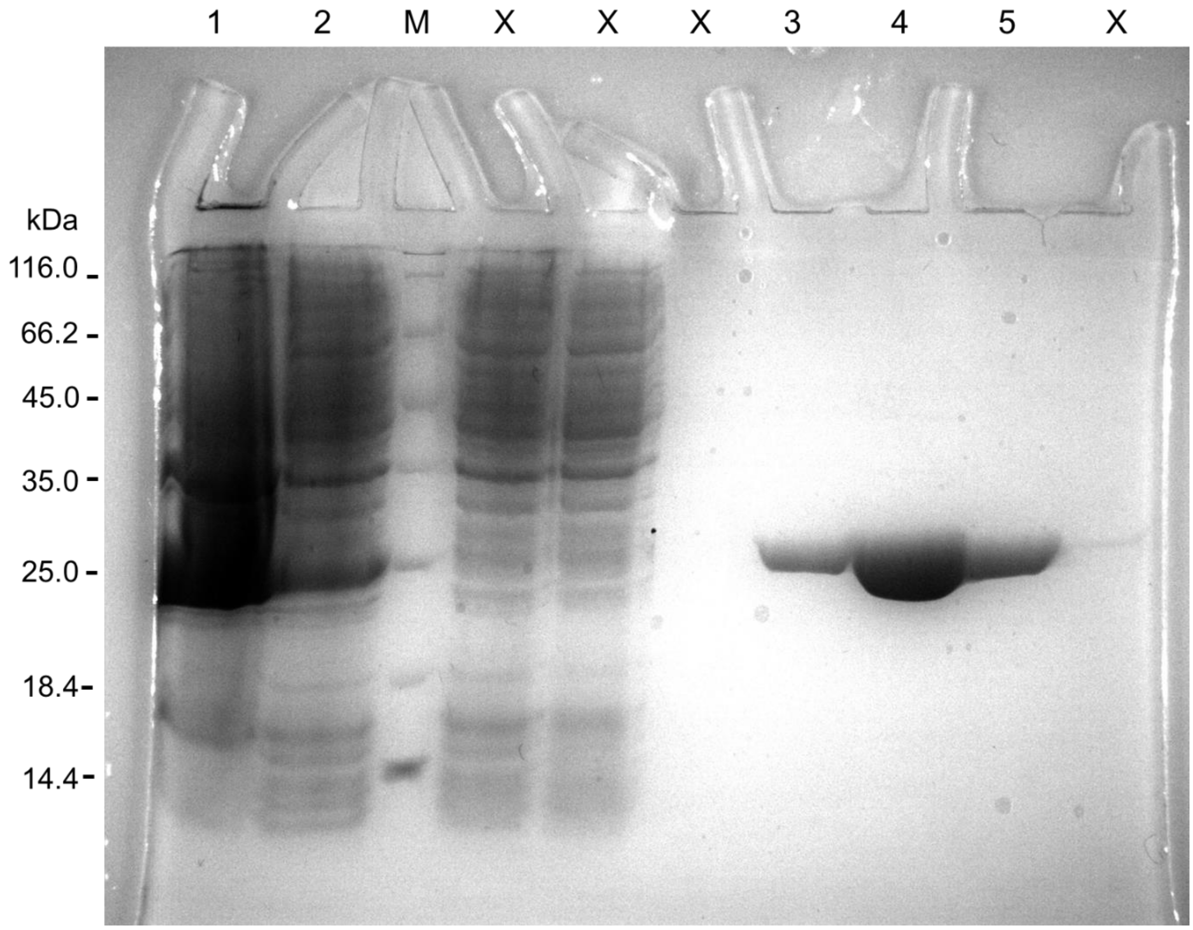

S1_raw_image_1. Raw gel image of protein molecular weight marker (first, top & bottom panels from the left) and BLEG-1 WT (second, top & bottom panels from the left) shown in Fig 11B. In this image, lane M: unstained protein marker (Thermo Fisher Scientific, USA) with size range of 14.4-116.0 kDa; lane 1: total cell extract (insoluble fraction); lane 2: total cell extract (soluble fraction); lane 3–5: Purified fractions. “X” refers to lanes not included in Fig 11B. The molecular weight of BLEG-1 WT is 26 kDa. Gel image was captured using the Syngene G:box F3 gel documentation system (Syngene, UK).

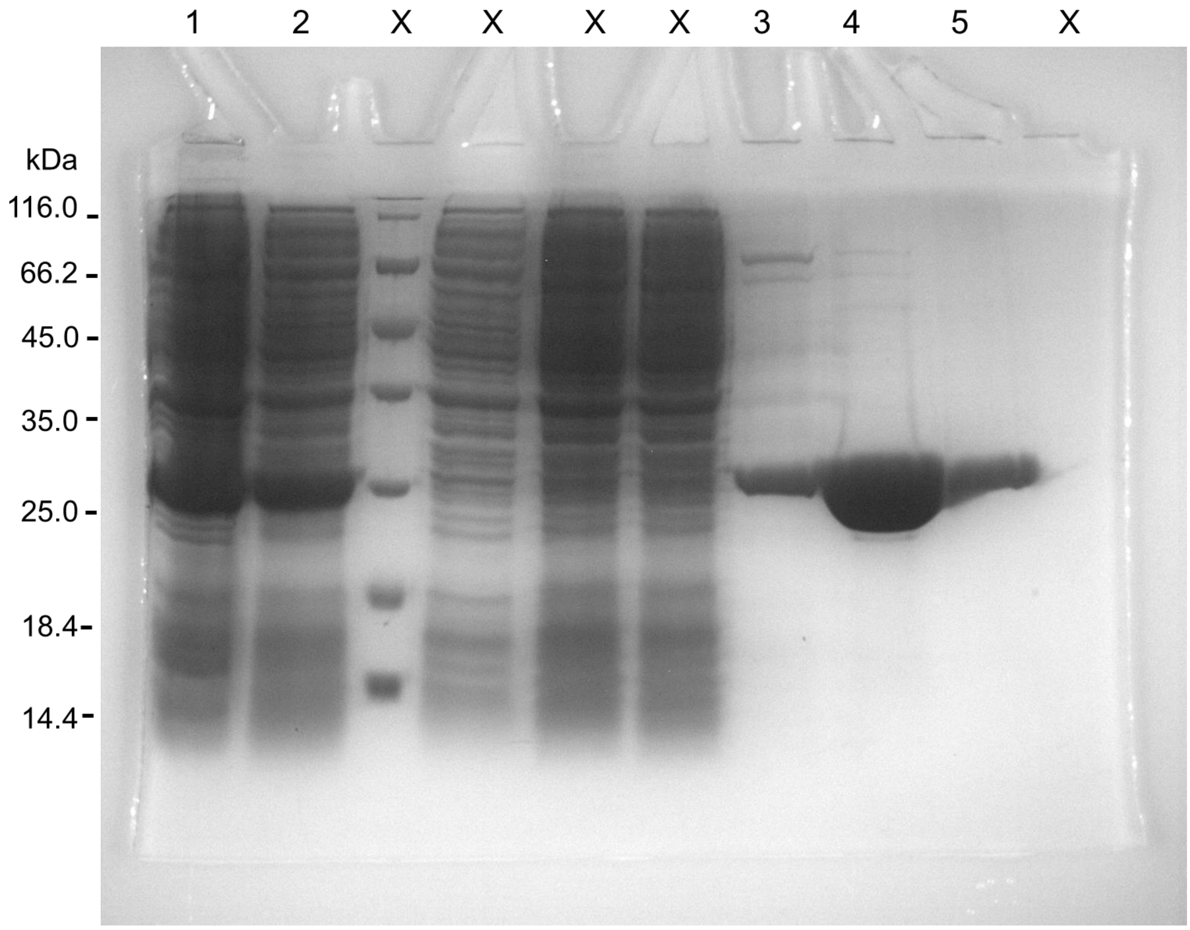

S1_raw_image_2. Raw gel image of BLEG-1 I10A (third, top & bottom panels from the left) shown in Fig 11B. In this image, lane 1: total cell extract (insoluble fraction); lane 2: total cell extract (soluble fraction); lane 3–5: Purified fractions. “X” refers to lanes not included in Fig 11B. The molecular weight of BLEG-1 I10A is 26 kDa. Gel image was captured using the Syngene G:box F3 gel documentation system (Syngene, UK).

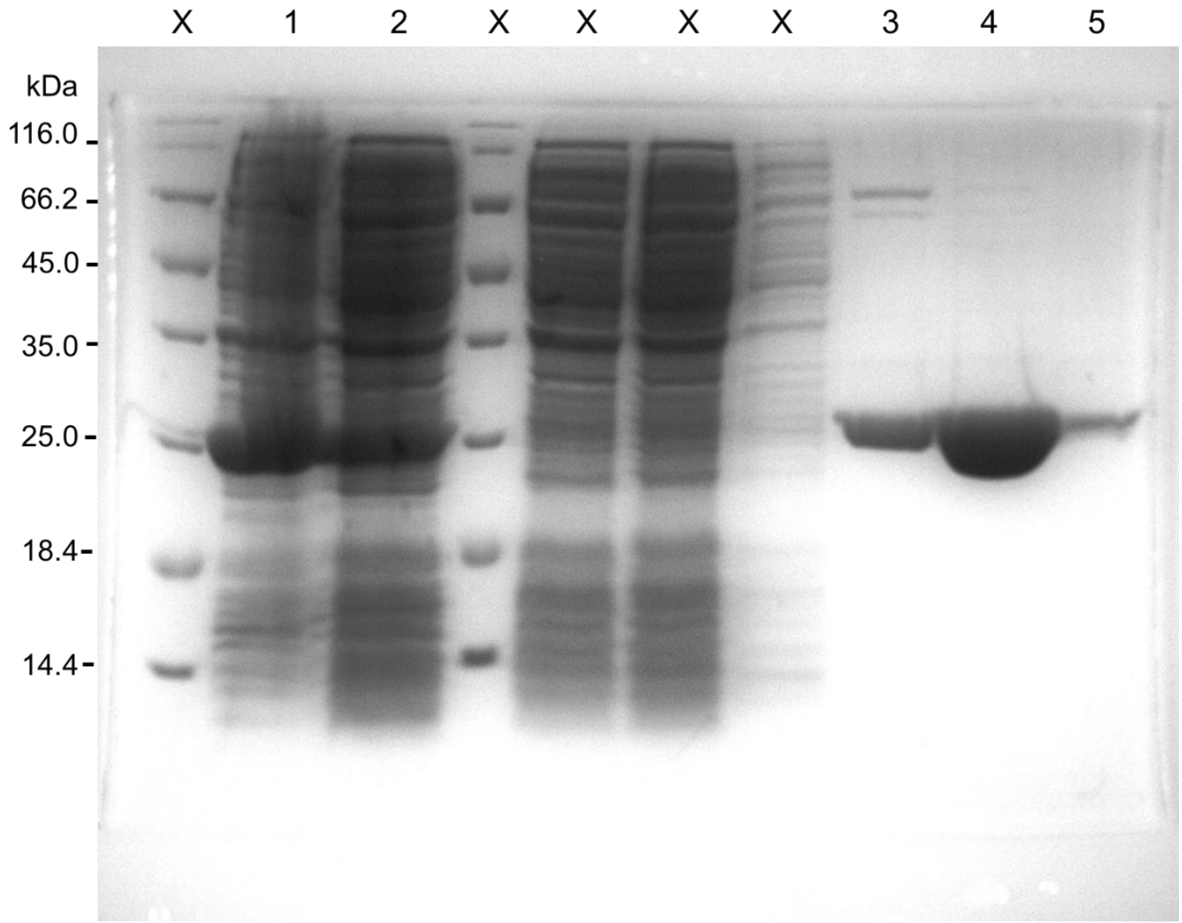

S1_raw_image_3. Raw gel image of BLEG-1 F57A (fourth, top & bottom panels from the left) shown in Fig 11B. In this image, lane 1: total cell extract (insoluble fraction); lane 2: total cell extract (soluble fraction); lane 3–5: Purified fractions. “X” refers to lanes not included in Fig 11B. The molecular weight of BLEG-1 F57A is 26 kDa. Gel image was captured using the Syngene G:box F3 gel documentation system (Syngene, UK).

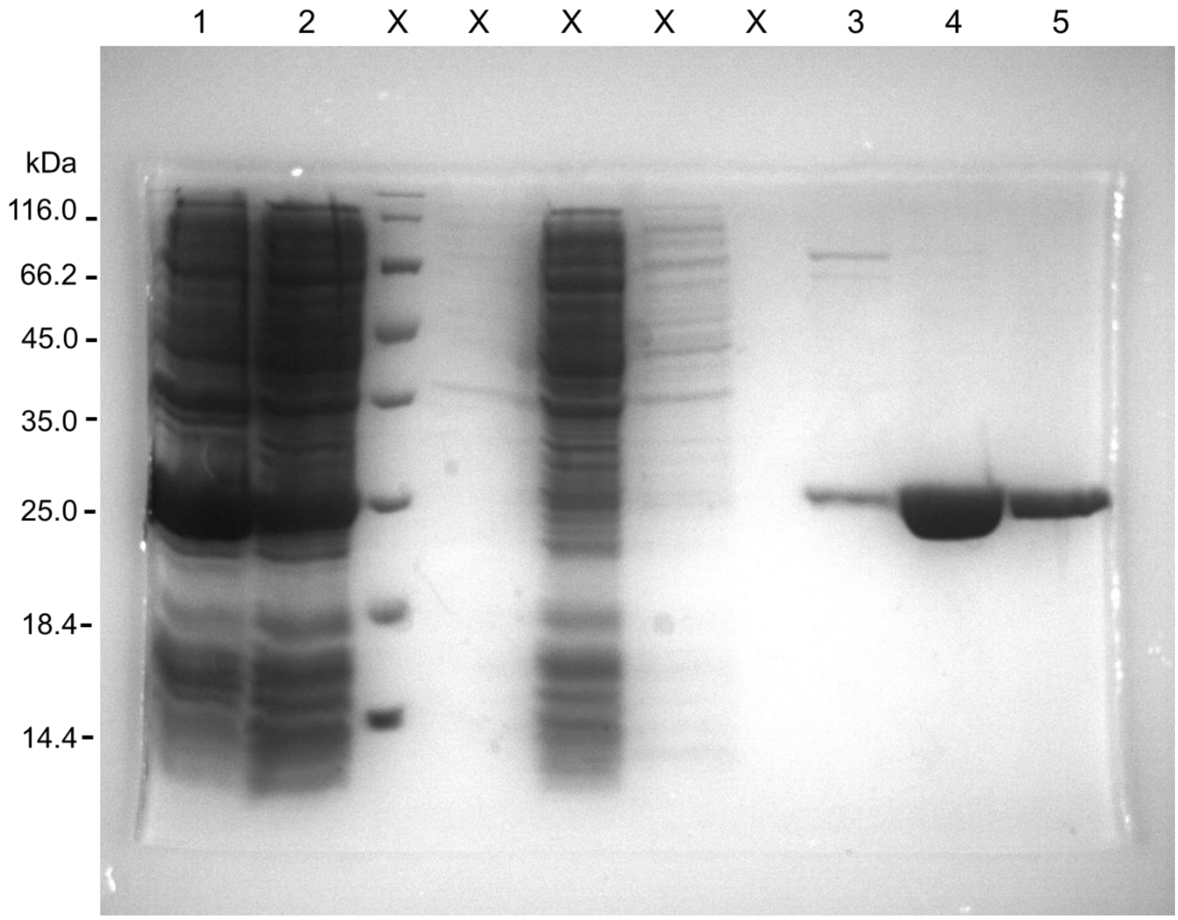

S1_raw_image_4. Raw gel image of BLEG-1 R94A (fifth, top & bottom panels from the left) shown in Fig 11B. In this image, lane 1: total cell extract (insoluble fraction); lane 2: total cell extract (soluble fraction); lane 3–5: Purified fractions. “X” refers to lanes not included in Fig 11B. The molecular weight of BLEG-1 R94A is 26 kDa. Gel image was captured using the Syngene G:box F3 gel documentation system (Syngene, UK).

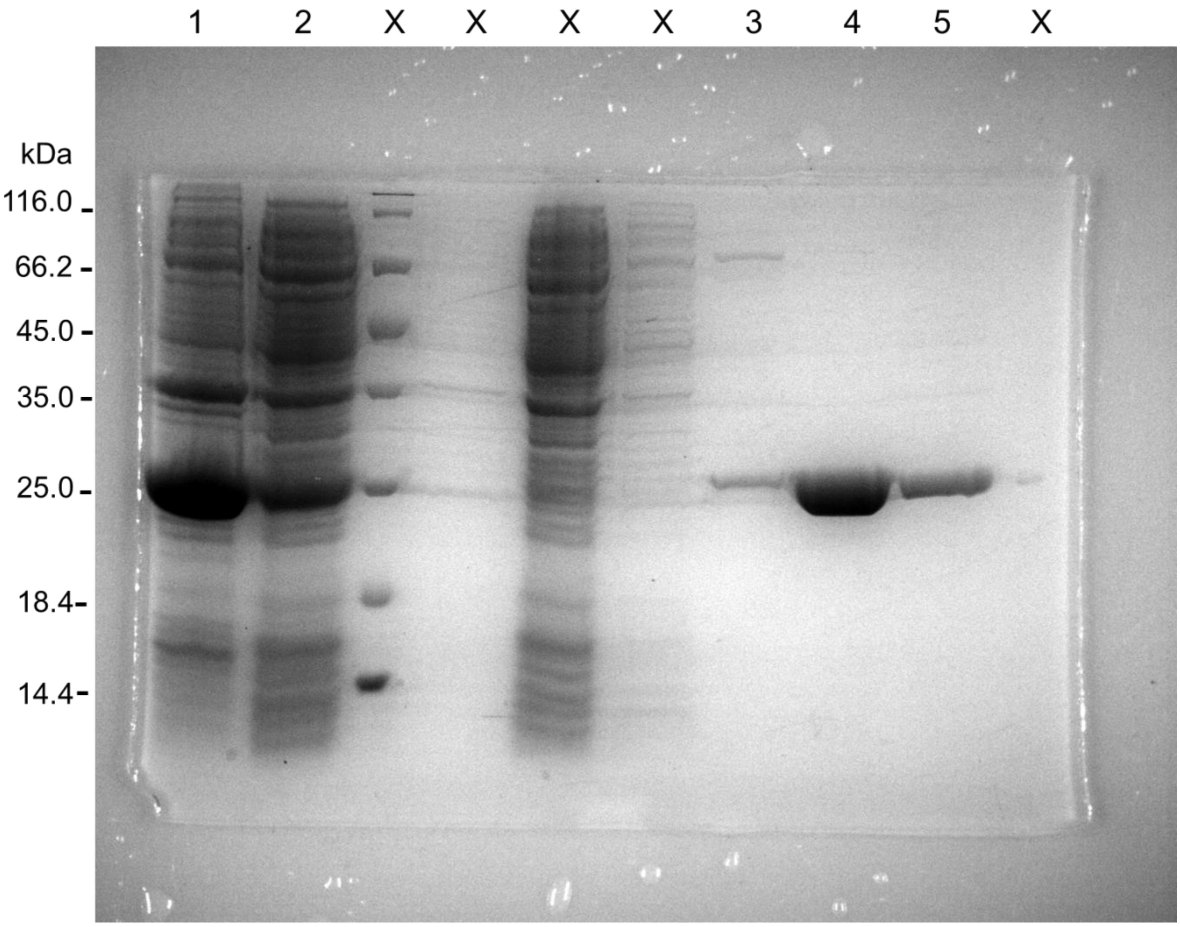

S1_raw_image_5. Raw gel image of BLEG-1 L95A (sixth, top & bottom panels from the left) shown in Fig 11B. In this image, lane 1: total cell extract (insoluble fraction); lane 2: total cell extract (soluble fraction); lane 3–5: Purified fractions. “X” refers to lanes not included in Fig 11B. The molecular weight of BLEG-1 L95A is 26 kDa. Gel image was captured using the Syngene G:box F3 gel documentation system (Syngene, UK).

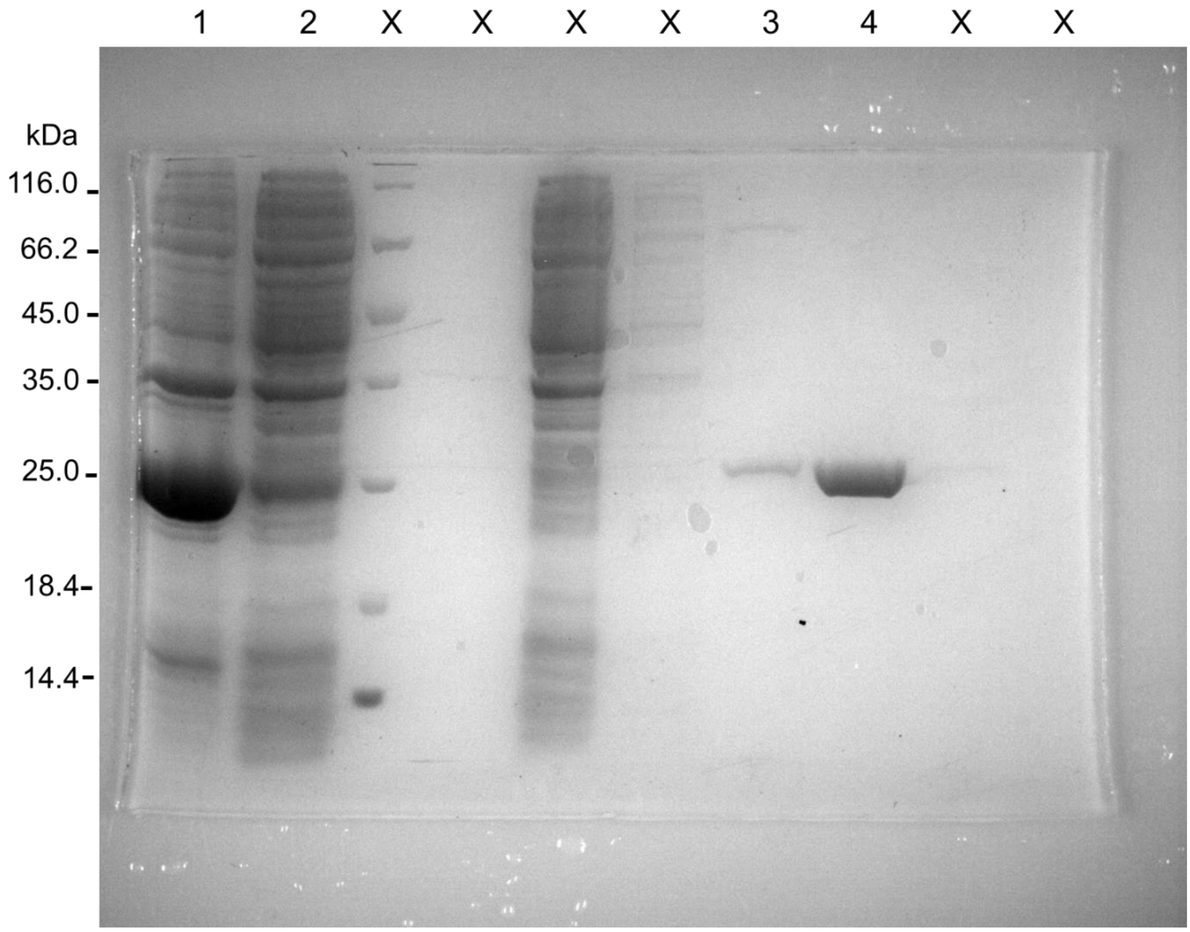

S1_raw_image_6. Raw gel image of BLEG-1 R159A (seventh, top & bottom panels from the left) shown in Fig 11B. In this image, lane 1: total cell extract (insoluble fraction); lane 2: total cell extract (soluble fraction); lane 3–4: Purified fractions. "X" refers to lanes not included in Fig 11B. The molecular weight of BLEG-1 R159A is 26 kDa. Gel image was captured using the Syngene G:box F3 gel documentation system (Syngene, UK).
